# ResR/McdR-regulated protein translation machinery contributes to drug resilience in *Mycobacterium tuberculosis*

**DOI:** 10.1038/s42003-023-05059-8

**Published:** 2023-07-11

**Authors:** Pramila Pal, Mohd Younus Khan, Shivani Sharma, Yashwant Kumar, Nikita Mangla, Prem S. Kaushal, Nisheeth Agarwal

**Affiliations:** 1 Translational Health Science and Technology Institute, NCR Biotech Science Cluster, 3rd Milestone, Faridabad–Gurgaon Expressway, Faridabad, 121001 Haryana India; 2 Jawaharlal Nehru University, New Mehrauli Road, New Delhi, 110067 Delhi India; 3 Regional Centre for Biotechnology, NCR Biotech Science Cluster, 3rd Milestone, Faridabad–Gurgaon Expressway, Faridabad, 121001 Haryana India

**Keywords:** Bacteriology, Molecular biology

## Abstract

Survival response of the human tuberculosis pathogen, *Mycobacterium tuberculosis* (Mtb) to a diverse environmental cues is governed through its versatile transcription regulatory mechanisms with the help of a large pool of transcription regulators (TRs). Rv1830 is one such conserved TR, which remains uncharacterized in Mtb. It was named as McdR based on an effect on cell division upon its overexpression in *Mycobacterium smegmatis*. Recently, it has been implicated in antibiotic resilience in Mtb and reannotated as ResR. While Rv1830 affects cell division by modulating the expression of *M. smegmatis whiB2*, the underlying cause of its essentiality and regulation of drug resilience in Mtb is yet to be deciphered. Here we show that ResR/McdR, encoded by *ERDMAN_2020* in virulent Mtb Erdman, is pivotal for bacterial proliferation and crucial metabolic activities. Importantly, ResR/McdR directly regulates ribosomal gene expression and protein synthesis, requiring distinct disordered N-terminal sequence. Compared to control, bacteria depleted with *resR/mcdR* exhibit delayed recovery post-antibiotic treatment. A similar effect upon knockdown of *rplN* operon genes further implicates ResR/McdR-regulated protein translation machinery in attributing drug resilience in Mtb. Overall, findings from this study suggest that chemical inhibitors of ResR/McdR may be proven effective as adjunctive therapy for shortening the duration of TB treatment.

## Abstract (web summary)

ResR/McdR regulates ribosomal activity and is pivotal for proliferation and crucial metabolic activities in *Mycobacterium tuberculosis*, thereby contributing to drug resilience in this pathogen.

## Introduction

Tuberculosis (TB) is an airborne communicable disease caused by *Mycobacterium tuberculosis* (Mtb), which caused nearly 1.5 million deaths worldwide in 2020^1^. Mtb is considered one of the most successful human pathogens, which can survive host arsenals efficiently. A tremendous survival capacity under varying environmental conditions can be attributed to the pathogen’s ability to differentially regulate the expression of various genes under diverse stresses imposed by the host cells^2–4^. The expression of a gene can be altered at the transcript level by a specific set of proteins called transcription regulators (TRs). TRs are characterized by the presence of DNA-binding motifs such as the helix-turn-helix (HTH) motif that enables them to recognize specific DNA sequences primarily in the 5’-untranslated region (5’-UTR) of a gene. TRs bind with the DNA sequence either alone or in association with partner proteins/chemical messengers, and subsequently modulate transcription. Therefore, TRs are crucial in shaping the pathogen’s transcriptional landscape and mounting a coordinated response to counter adverse environmental stimuli. TRs are also the nodal point of multiple signaling pathways^5^ and are perceived as one of the major drug targets for screening small molecule inhibitors^6^.

Sequence analysis of the Mtb genome reveals the presence of 214 genes encoding for proteins with DNA-binding motifs, suggestive of their putative involvement in transcription^7^. Earlier studies have shown that ~70% of the Mtb genome undergoes regulation upon overexpression of the prospective transcriptional regulatory proteins^7,8^. TRs are supposed to be critical for the regulation of metabolic pathways in the pathogen during the changing environmental conditions, both extracellularly as well as in the host. One of the factors determining bacterial virulence is its ability to undergo a non-replicative dormant state, which is achieved by the metabolic shutdown with the help of TRs^9^. Importantly, TRs are also responsible for alleviating dormancy by affecting bacterial cell wall biosynthesis and central metabolism^10^. In addition, TRs also play an important role in controlling bacterial response to hypoxia^11^, oxidative burst^12^, starvation^13^, acid stress^14^, and a variety of other conditions, which together imply the importance of TRs in the pathophysiology of Mtb.

Genome-wide screening by transposon site hybridization (TraSH) has identified 12 TRs that might be essential for in vitro growth of the virulent Mtb H_37_Rv. Rv1830, present ubiquitously across actinobacteria including different mycobacterial species, is one such essential TR^8^ whose role in Mtb pathophysiology is poorly understood. As overexpression of Rv1830 in the heterologous host *M. smegmatis*, a fast-growing avirulent mycobacterium, causes altered expression of genes involved in cell division and DNA repair mechanisms, it was annotated as McdR (for *m*ycobacterial cell division *r*egulator)^15^. Interestingly, a recent study has identified *Rv1830* as one of the genes which exhibit a higher ratio of nonsynonymous to synonymous mutations across more than 50,000 clinical isolates of Mtb, suggestive of its adaptive selection during host infection. The *Rv1830* mutants resume growth at a faster rate compared to the wild-type strain following exposure to antibiotics, despite no change in bacterial susceptibility to drugs. Based on its involvement in modulating the post-antibiotic effect (PAE), an event in which bacterial growth is delayed following brief exposure to antimicrobials leading to altered antibiotic resilience, *Rv1830* was annotated as *resR*^16,17^. While overexpression of this gene affects cell division via regulation of *whiB2* expression in *M. smegmatis*, it remains to understand the underlying cause of *resR/mcdR* essentiality and regulation of drug resilience in Mtb.

Herein we show that *ERDMAN_2020*, an orthologue of *resR/mcdR* in virulent Mtb Erdman strain, is vital for replication of the pathogen in the synthetic culture medium as well as during infection of animals. Whole genome transcriptional profiling of Mtb depleted with *resR/mcdR* demonstrates massive transcriptional reprogramming with the perturbation of ~800 genes including those associated with important biological activities. Further, we show that ResR/McdR plays a critical role in protein translation in Mtb through direct regulation of *rplN* operon genes that encode for various large and small subunits of the ribosome. Remarkably, CRISPRi silencing of *resR/mcdR* as well as *rplN* genes results in an increased PAE leading to slower recovery of bacteria following exposure to antibiotics, when compared with the control strain. Taken together, these results suggest that Mtb resilience to antibiotics is likely controlled by ResR/McdR-mediated regulation of protein translation machinery.

## Results

### ResR/McdR is a MerR family of transcription regulators which is expressed at all stages of growth in Mtb

Sequence analysis reveals that the ResR/McdR belongs to a MerR family of transcription regulators, which is highly conserved across different actinobacteria (Supplementary Fig. 1) including Mtb complex bacteria (Supplementary Fig. 2). ResR/McdR homologs from mycobacteria exhibit ≥90% sequence similarity, whereas those from actinobacteria show 50–80% identical residues. In silico prediction of ResR/McdR conformation by d2p2 tool^18^ reveals the presence of an HTH domain between 65–137 amino acid residues, whereas the N-terminal (1–48 amino acid) and the C-terminal (201–225) regions are highly disordered (Supplementary Fig. 3a). Notably, ResR/McdR is exclusive to actinobacteria and the homologous sequences were not found by BLASTP search in any other bacteria, archaea, or eukaryotes. Sequence conservation is observed primarily in the HTH region, whereas the adjacent sequences are variable across different organisms (Supplementary Figs. 1 and 2). Akin to the MerR family of TRs, which tend to adopt a dimeric conformation,^19,20^ the purified 6× His-tagged ResR/McdR protein also shows the presence of oligomers, as assessed by size exclusion chromatography (Supplementary Fig. 3b).

Subsequent analysis of the *resR/mcdR* locus shows the presence of two other genes, namely *ERDMAN_2021* and *gcvB* (Supplementary Fig. 4a). While the PCR amplification of the *resR/mcdR-ERDMAN_2021* and *resR/mcdR_gcvB* junction sequences using the genomic DNA (gDNA) as template yields the desired amplicons of 815 bp and 1000 bp, respectively (Supplementary Fig. 4b, lanes 1 and 5), we fail to observe amplification of the corresponding sequences with the complementary DNA (cDNA) template (Supplementary Fig. 4b, lanes 2 and 6). Importantly, a sequence of 678 bp within *resR/mcdR* ORF can be amplified with the same cDNA preparation (Supplementary Fig. 4b, lanes 3 and 7) thus assuring the quality of the cDNA used for PCR amplification in the above reactions. These results suggest that *resR/mcdR* expression is independent of the other two genes downstream to it.

Next, we analyzed the expression of ResR/McdR protein at different growth stages *viz*., lag (OD_600_ = ~0.1), mid-log (OD_600_ = 0.5–1.0), late-log (OD_600_ = ~3.0) and stationary (OD_600_ ≥ 3.0) phases (Supplementary Fig. 4c) by anti-ResR/McdR immunoblotting. Whole cell lysates prepared from different bacterial samples were quantitated and an equal amount of lysates were transferred to the nitrocellulose membrane. The membrane was stained with the ponceau stain before incubation with the primary antibodies, to ascertain a similar loading profile (Supplementary Fig. 4d). Subsequent analysis of ResR/McdR signals on the immunoblot reveals its expression at all growth stages, thus suggesting the constitutive requirement of ResR/McdR in Mtb.

### ResR/McdR is important for in vitro proliferation of Mtb

**Fig. 1:**
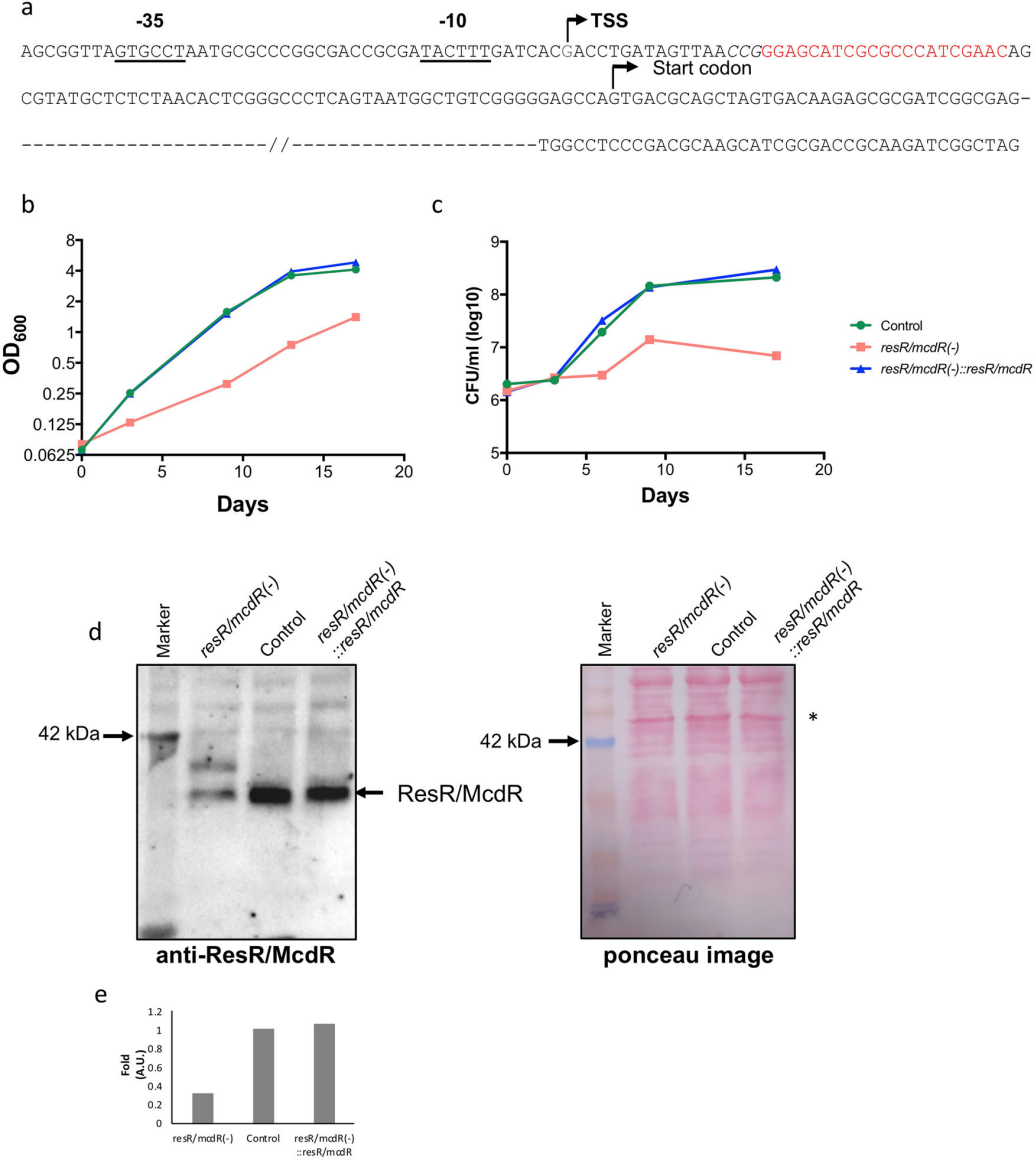
*resR/mcdR* is essential for in vitro growth of Mtb. **a** Analysis of the *resR/mcdR* promoter sequence. Transcription start site (TSS) and SigA-recognition sequences at the −10 and −35 positions in the 5’-UTR of *resR/mcdR*, as identified by 5’-RACE, are underlined. Sequence in italics represents the complementary sequence of protospacer adjacent motif (PAM) in the non-template strand, and the gRNA-hybridization sequence is marked red. **b**, **c** Effect of *resR/mcdR* depletion on in vitro growth of Mtb. In vitro growth of control, mutant (*resR/mcdR(−)*) and the complemented (*resR/mcdR(−)::resR/mcdR*) strains of Mtb was analyzed after treatment with 50 ng/ml ATc by estimating OD_600_ (**b**) and by CFU plating (**c**) at successive time points. **d** Analysis of ResR/McdR expression by immunoblotting. Shown is the anti-ResR/McdR immunoblot of whole cell lysates from the *resR/mcdR(−)*, control, and *resR/mcdR(−)::resR/mcdR* strains of Mtb. Equal loading of samples is confirmed by Ponceau staining of the membrane before probing with the anti-ResR/McdR antibodies. **e** Analysis of ResR/McdR signal intensity. Signal intensities were analyzed by densitometric scanning of the anti-ResR/McdR blot in **d** using ImageJ software after normalization with the intensity of a 50 kDa band in the respective lanes in the ponceau-stained blot (marked by an asterisk in **d**). Data represent mean values from multiple (*n* = 3) measurements in **b** and multiple (*n* = 2) biological repeat experiments in **c**.

Although *resR/mcdR* is predicted essential by TraSH screen^21^, experimental validation is yet to be performed to discern its genetic requirement in the virulent Mtb Erdman. Conditional knockdown of *resR/mcdR* was achieved in Mtb Erdman by using the CRISPRi approach, as described in Methods. The transcriptional start site (TSS) in the 5’-UTR was determined by 5’-rapid amplification of cDNA end (5’-RACE) technique^22^, which reveals that the transcription of this gene starts from a G residue situated at 90nt upstream to the start codon (Fig. 1a and Supplementary Fig. 5). Further analysis of the *resR/mcdR* promoter sequence shows the presence of conserved residues in the −10 (5’-TACTTT-3’) and −35 (5’-GTGCCT-3’) motifs, as reported earlier^23^. Based on these results, a PAM sequence 5’-CGG-3’ was mapped in the template strand between +17 to +15 position from the TSS, and a 21nt guide sequence was designed such that the resulting guide RNA (gRNA) would hybridize with the non-template strand in the promoter region of *resR/mcdR* (Fig. 1a).

Treatment of the *resR/mcdR* knockdown strain [annotated as *resR/mcdR(−)*] with different concentrations of ATc for 4 days results in an ATc dose-dependent suppression of *resR/mcdR* transcripts. A significant reduction in *resR/mcdR* transcripts by 85% (*p* < 0.0005) is achieved with 50 ng/ml ATc, which also leads to a concomitant increase in *dcas9* expression by ~40-fold (*p* < 0.05) in comparison to the untreated control (Supplementary Fig. 6a, b). The subsequent increase in ATc concentration does not improve the silencing efficiency. Remarkably, a gradual decrease in *resR/mcdR* transcript levels in *resR/mcdR(−)* upon treatment with different doses of ATc causes a significant decline in bacterial growth, as analyzed by OD_600_ and CFU estimations (Supplementary Fig. 6c, d).

After these preliminary observations, in vitro growth of *resR/mcdR(−)* was monitored at regular intervals in the presence of 50 ng/ml ATc by estimating both OD_600_ (Fig. 1b and Supplementary Data 1) as well as bacterial CFU counts (Fig. 1c and Supplementary Data 1). Bacteria harboring pDcas9 were simultaneously used as control. Our results show an increase in the CFU of *resR/mcdR(−)* by 1.75-, 1.97-, 9.27-, and 5.30-fold on days 3, 6, 9, and 17, respectively, compared to day 0 CFU. In contrast, while the control strain exhibits a similar trend during the initial time point of day 3 with a marginal increase in CFU by 1.2-fold, bacterial count is drastically increased by 9.51-, 71.57- and 105.88-fold on days 6, 9, and 17, when compared with day 0 CFU (Fig. 1c and Supplementary Data 1). To ascertain that weak proliferation of Mtb is indeed due to loss of *resR/mcdR* expression, growth was monitored in the presence of the wild-type *resR/mcdR*, which was simultaneously expressed under ATc-inducible promoter in the *resR/mcdR(−)* knockdown strain. As presented in Fig. 1b, c, expression of wild-type *resR/mcdR* restored the attenuated growth phenotype of the *resR/mcdR(−)* strain, thus confirming the indispensable requirement of this gene for the extracellular growth of Mtb. Noteworthy to mention, loss of *resR/mcdR* in the knockdown strain and its restoration upon expression of wild-type copy in the *resR/mcdR(−)* is validated by anti-ResR/McdR immunoblotting of the whole cell lysates on day 6 post-ATc treatment. As can be seen in Fig. 1d, e, silencing of *resR/mcdR* by CRISPRi results in ~70% reduction in the expression of ResR/McdR in the mutant strain, which is restored nearly to its level in the control upon ATc-inducible expression from the extrachromosomal plasmid DNA (Fig. 1d, e, Supplementary Fig. 7 and Supplementary Data 1). Notably, modulation of *resR/mcdR* leads to a minor increase in the median cell length of bacteria, as observed by scanning electron microscopy. While the control strain exhibits the median length of 1.656 ± 0.037 µm, cell length increases by ~15% to 1.914 ± 0.045 µm (*p* < 0.05) upon suppression of *resR/mcdR* (Supplementary Fig. 8), which is in an agreement with the previous report^15^.

### *resR/mcdR* is required for intracellular proliferation of Mtb in the host

**Fig. 2:**
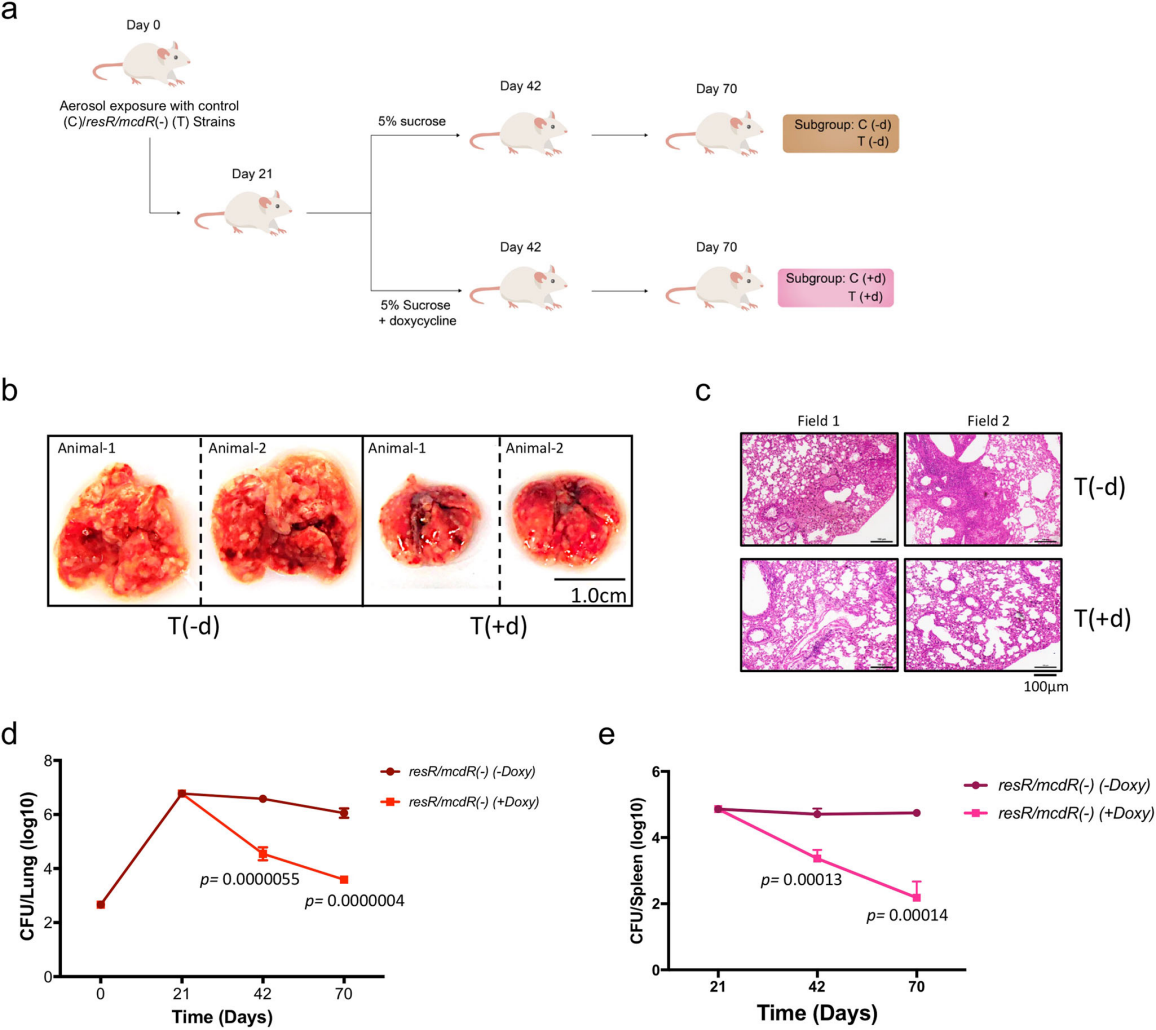
*resR/mcdR* is critical for the intracellular proliferation of Mtb. **a** Schematic of mouse infection. Mice were infected by aerosol route with the control (depicted as ‘C’) and the *resR/mcdR(−)* (depicted as ‘T’) strains of Mtb Erdman. After 21 days of infection, mice were divided into two groups: one group of mice receiving only 5% sucrose (-d) and others receiving doxycycline in 5% sucrose (+d). Intracellular bacterial load was determined by CFU plating of lungs and spleen homogenates at the indicated time points. **b** Gross pathology of lungs. Shown are the images of lungs obtained from T(-d) and T(+d) groups of mice after 49 days of treatment (i.e., day 70 post-infection). Scale bar is shown for size reference. **c** Histopathology of lungs from Mtb-infected mice. Histopathology was performed by H&E staining of a section of lungs from the T(-d) and the T(+d) group of mice after 49 days of treatment (i.e., day 70 post-infection). Representative low-magnification micrographs of sections of lungs from both groups are shown for comparison. Scale bar is shown for size reference. **d**, **e** Effect of doxycycline treatment on intracellular survival of *resR/mcdR(−)* strain of Mtb. Intracellular survival of the *resR/mcdR(−)* mutant strain was determined by estimating the bacterial load in lungs (**d**) and spleen (**e**) at the respective time points by CFU enumeration. Data represent mean ± s.d. (shown by error bars) values from multiple (*n* = 4) animals in **d**–**e**. *p* values in **d**, **e** were obtained at the indicated time points after comparison with day 21, as described in Methods.

Since bacteria remain viable under the extracellular culture conditions upon *resR/mcdR* silencing, we next examined the impact of *resR/mcdR* depletion on the intracellular growth of the Mtb pathogen in the host. Infection of BALB/c mice was set up with empty vector control and *resR/mcdR(−)* strains through aerosol inhalation, as reported in the Methods. After infection for three weeks, both control and *resR/mcdR(−)* infected mice (annotated as C and T, respectively; Fig. 2a) were divided into 2 groups, one receiving 5% sucrose in drinking water [C(-d) or T(-d)] and other 1 mg/ml doxycycline in the drinking water containing 5% sucrose [C(+d) or T(+d)]. Doxycycline treatment was administered to achieve silencing of *resR/mcdR* in the pathogen infecting T(+d) group of animals, whereas the same in C(+d) group was used as control. The doxycycline dose was carefully chosen based on a previous study that reports no adverse effect on the health of mice and is sufficient to regulate TetR-dependent expression of transcripts in Mtb during infection^24^. Gross pathology of infected lungs, as well as histopathology of lung sections obtained at day 70 post-infection (day49 post-doxycycline treatment), indicate that the disease burden is severely attenuated in T(+d) group in comparison to the T(-d) group of mice (Fig. 2b, c). Lungs of the T(-d) group of mice exhibit a large number of granulomatous lesions compared to those obtained from T(+d) group. Moreover, the enumeration of bacterial CFU at regular intervals reveals a significant drop in CFU of *resR/mcdR(−)* in both lungs and spleens following the doxycycline treatment. Treatment of mice with 1 mg/ml doxycycline results in 2.03 log_10_-fold reduction (*p* < 0.00005) in the intracellular CFU counts of *resR/mcdR(−)* after 21 days, and 2.46 log_10_-fold reductions (*p* < 0.0000005) after 49 days of treatment (Fig. 2d and Supplementary Data 1). Similar to the lungs, a sharp decline in CFU counts are also observed in the spleen of the T(+d) group of mice, whereas the bacterial load remains stable in the spleen of the T(-d) group of animals (Fig. 2e and Supplementary Data 1). Estimation of intracellular bacterial load in the spleen of mice from the T(+d) group reveals a reduction by 1.34 log_10_-fold (*p* < 0.0005) and 2.56 log_10_-fold (*p* < 0.0005) after 21- and 49 days of doxycycline treatment, respectively (Fig. 2e and Supplementary Data 1). Noteworthy to mention that the intracellular bacterial load remains unaffected by doxycycline treatment of mice infected with the control strain of Mtb (Supplementary Fig. 9), which confirms that reduction in disease burden upon infection with *resR/mcdR(−)* is indeed due to doxycycline-mediated silencing of the *resR/mcdR* gene. Overall, the above results establish that *resR/mcdR* is indispensable not only for extracellular proliferation but also for the survival of the pathogen in the host.

### Silencing of *resR/mcdR* expression in Mtb leads to massive transcriptional reprogramming

**Fig. 3:**
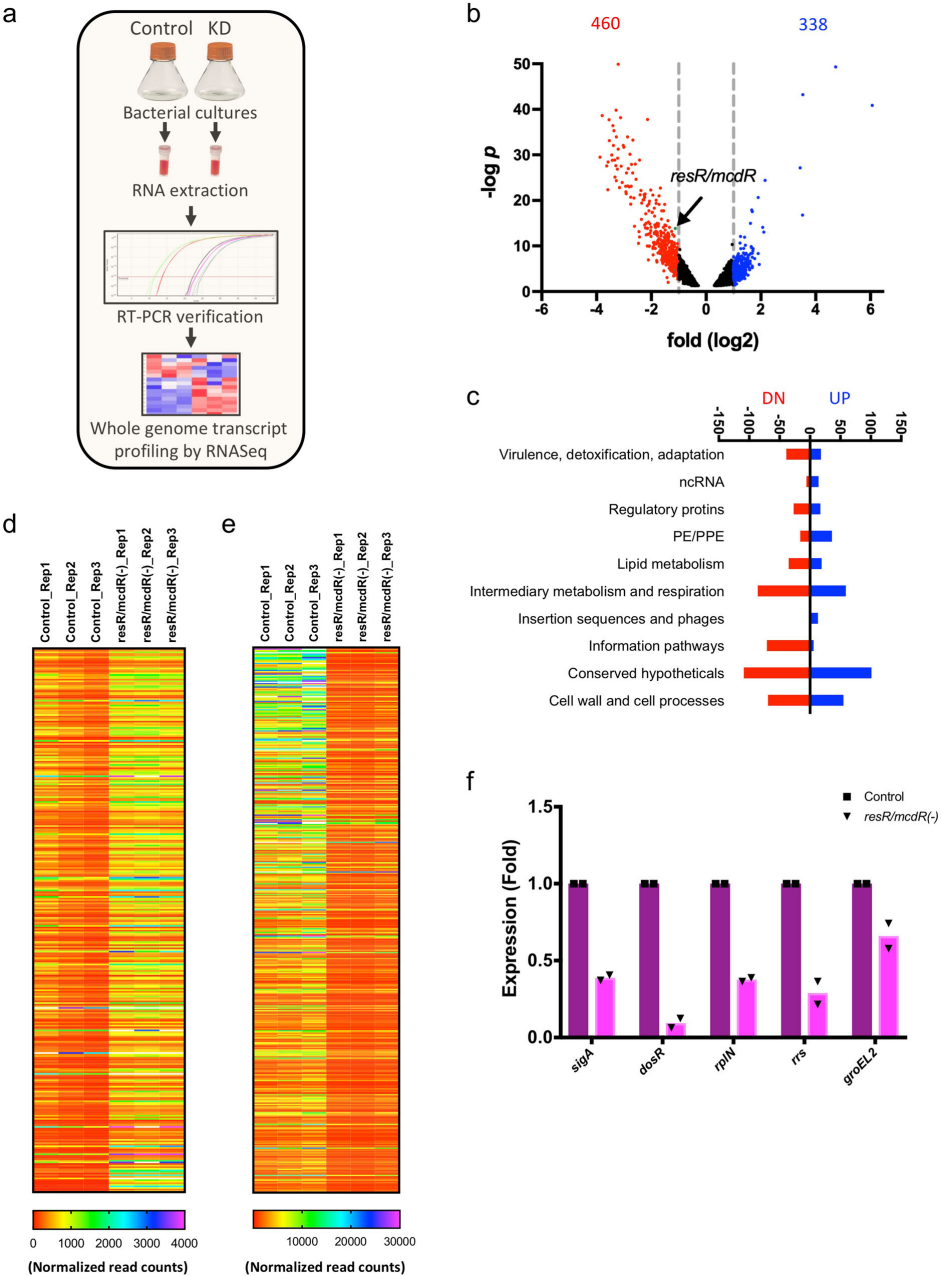
Effect of *resR/mcdR* depletion on the whole genome transcriptional profile of Mtb. **a** Schematic of the strategy used for whole genome transcriptional profiling of Mtb. Briefly, bacterial cultures of the empty vector control and *resR/mcdR(−)* (KD) strains were treated with 50 ng/ml ATc for four days, followed by extraction of RNA. After verification of *resR/mcdR* silencing in the KD by qRT-PCR, samples were processed for RNA sequencing as described in the text. **b** Volcano plot of differentially expressed genes in *resR/mcdR(−)*. Shown is the distribution of differentially expressed genes via log2 (fold-change) and >1.3 −log *p* values. Broken vertical lines represent the cutoff of ≥1 log2 fold-change. Genes undergoing downregulation are represented by red dots, and those showing upregulation in *resR/mcdR(−)* are marked with blue dots. The status of *resR/mcdR* expression is highlighted by an arrow. Data represent fold change in read counts between *resR/mcdR(−)* and control strains from three biological replicates. **c** Functional categorization of differentially expressed genes. Shown is the butterfly chart for the distribution pattern of genes that are perturbed in *resR/mcdR(−)* according to their function. Different functional categories are defined according to classification by the Mycobrowser database (https://mycobrowser.epfl.ch/genes/). **d**, **e** Status of differentially accumulated transcripts. Heat maps represent transcripts showing accumulation (**d**) or suppression (**e**) upon *resR/mcdR* silencing. **f** Validation of RNAseq data. RNAseq data were verified by qRT-PCR analysis of select genes, using specific primer pairs (Supplementary Data 4). Fold change in expression of the respective transcripts was obtained after normalization with the level of a control gene *htpG*, which remains constant in both strains. Data represent mean values from multiple (*n* = 2) biological repeats.

To gain an insight into the effect of *resR/mcdR* silencing on bacterial viability, both the control and the *resR/mcdR(−)* of Mtb Erdman were subjected to the whole genome transcriptional analysis by RNA sequencing (RNASeq) by following a scheme as outlined in Fig. 3a. Total RNAs were extracted from both strains at the early time point of 4 days post-ATc treatment to minimize the growth defect due to *resR/mcdR* silencing. RNA samples, prepared from three independent sets, were subjected to RNASeq, which reveals significant variance between the control and the *resR/mcdR(−)* groups, as determined by the principal component analysis. Interestingly, ~800 genes equivalent to ~20% of the entire gene pool of Mtb exhibit differential expression by ≥2.0-fold change (*p* < 0.05) due to a partial reduction in the expression of *resR/mcdR* (Fig. 3b and Supplementary Data 2). While the expression of 460 genes is reduced, 338 genes are upregulated upon depletion of *resR/mcdR* in Mtb Erdman (Fig. 3b). A thorough investigation of RNASeq data reveals that depletion of *resR/mcdR* primarily affects the expression of genes associated with intermediary metabolism and respiration, information pathways, and cell wall and lipid metabolism along with a large number of hypothetical genes (Fig. 3c and Supplementary Data 1). Importantly, the expression pattern of both the down- and upregulated genes is highly consistent across the biological replicates (Fig. 3d, e) that are verified by qRT-PCR of a few representative genes using specific primers sets (Fig. 3f and Supplementary Data 1). Together, these results indicate that Mtb undergoes significant transcriptional reprogramming in response to the downregulation of *resR/mcdR*.

### Depletion of *resR/mcdR* in Mtb perturbs cellular metabolism

**Fig. 4:**
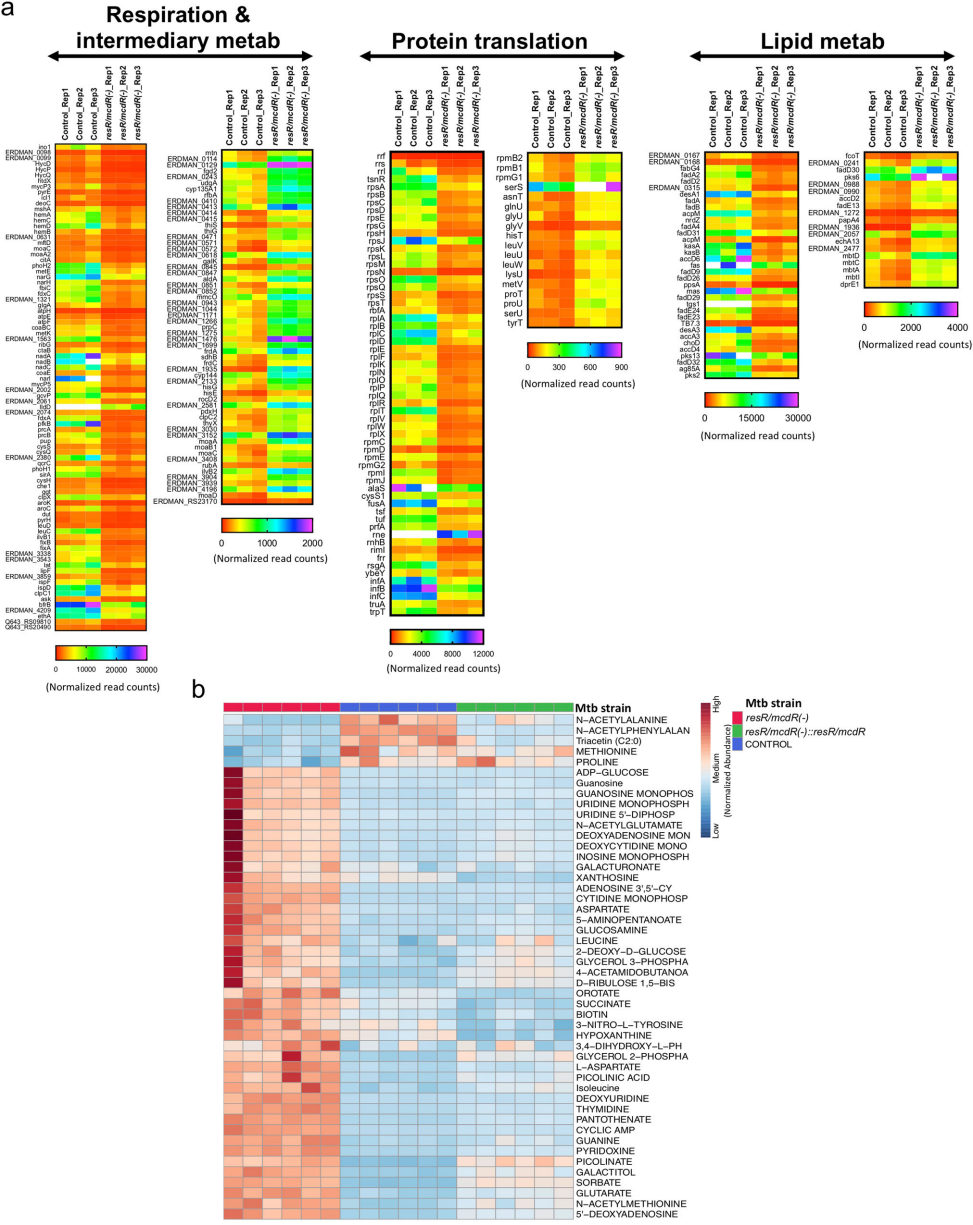
Effect of *resR/mcdR* depletion on metabolic profile of Mtb. **a** Heatmap representation of the expression levels of different metabolic genes. Genes associated with respiration, protein translation and lipid metabolism exhibiting differential expression by ≥2-fold (*p* < 0.05) in three biological repeat experiments are shown. **b** Heatmap analysis of metabolites. Level of different metabolites was estimated in the empty vector control (blue), *resR/mcdR(−*) (red) and *resR/mcdR(−)::resR/mcdR* (green) strains of Mtb mc^2^ 7902 by LC-MS/MS. The heatmap represents normalized abundance of metabolites that are modulated by ≥1.3-fold (*p* < 0.05) in *resR/mcdR(−)* compared to control and *resR/mcdR(−)::resR/mcdR* complemented strains across six biological repeats.

As described above, depletion of *resR/mcdR* primarily modulates the expression of genes that are associated with core metabolic activities of Mtb such as intermediary metabolism and respiration (*n* = 146), protein translation (*n* = 78), and lipid metabolism (*n* = 54) (Figs. 3c and 4a, and Supplementary Data 2). Apart from these, several other genes associated with essential metabolic pathways such as transcription, DNA replication and repair, membrane transport, protein folding, and degradation, are also modulated in the knockdown strain (Supplementary Data 2).

To further examine if the lethality of *resR/mcdR* knockdown is indeed due to perturbation of these metabolic pathways, we analyzed the status of polar as well as non-polar metabolites in the control, mutant, and the complemented strains of Mtb with the help of LC-MS/MS, as described earlier^25^. A total of 171 metabolites were identified from the six biological replicates, out of which 42 exhibit accumulation and 5 are depleted by ≥1.3-fold (*p* < 0.05) in the *resR/mcdR(−)* strain compared to their levels in the control. Importantly, the level of all of these metabolites is complemented upon the expression of another copy of *resR/mcdR* in the *resR/mcdR(−)* knockdown strain, thus confirming the specific effect of *resR/mcdR* silencing on these metabolites (Fig. 4b).

A careful examination of the metabolic profile of these strains further suggests that ~94% of differentially regulated metabolites in *resR/mcdR(−)* belong to three functional categories, *viz*., protein synthesis (*n* = 17), nucleotide metabolism (*n* = 15) and carbon metabolism (*n* = 12). The *resR/mcdR(−)* strain exhibits accumulation of several amino acids (aspartate, isoleucine, L-aspartate and leucine), amino acid derivatives (N-acetylglutamate and N-acetylmethionine), modified forms of amino acids (5-aminopentanoate, 3-nitro-L-tyrosine, 3,4-dihydroxy-L-phenylalanine and 4-acetamidobutanoate), and metabolic products of a few amino acids such as tryptophan (picolinic acid and picolinate) and β-alanine (pantothenate). Moreover, the level of a few amino acids (methionine and proline) and amino acid derivatives (*N*-acetylalanine and *N*-acetylphenylalanine) is found to be reduced in *resR/mcdR(−)* strain of Mtb. In addition to these, nine metabolites involved in purine biosynthesis (5’-deoxyadenosine, cyclic AMP, dAMP, guanine, guanosine, GMP, hypoxanthine, IMP, and xanthosine) and six metabolites associated with pyrimidine biosynthesis (CMP, dCMP, deoxyuridine, orotate, thymidine, UMP) exhibit significant accumulation, thus indicating perturbation of the nucleotide metabolic pathways upon *resR/mcdR* depletion. We find that depletion of *resR/mcdR* alters the level of several key metabolites that are associated with the central carbon metabolic pathway such as ADP-glucose, galactitol, galacturonate, glutarate, succinate, glucosamine, glycerol 2-phosphate, glycerol 3-phosphate, pantothenate, and D-ribulose 1,5-bisphosphate that exhibit accumulation in *resR/mcdR(−)* compared to their levels in the control bacteria (Fig. 4b). Overall, these results demonstrate a critical requirement for ResR/McdR in Mtb complex bacteria.

### Expression of ResR/McdR is pivotal for protein translation in Mtb

**Fig. 5:**
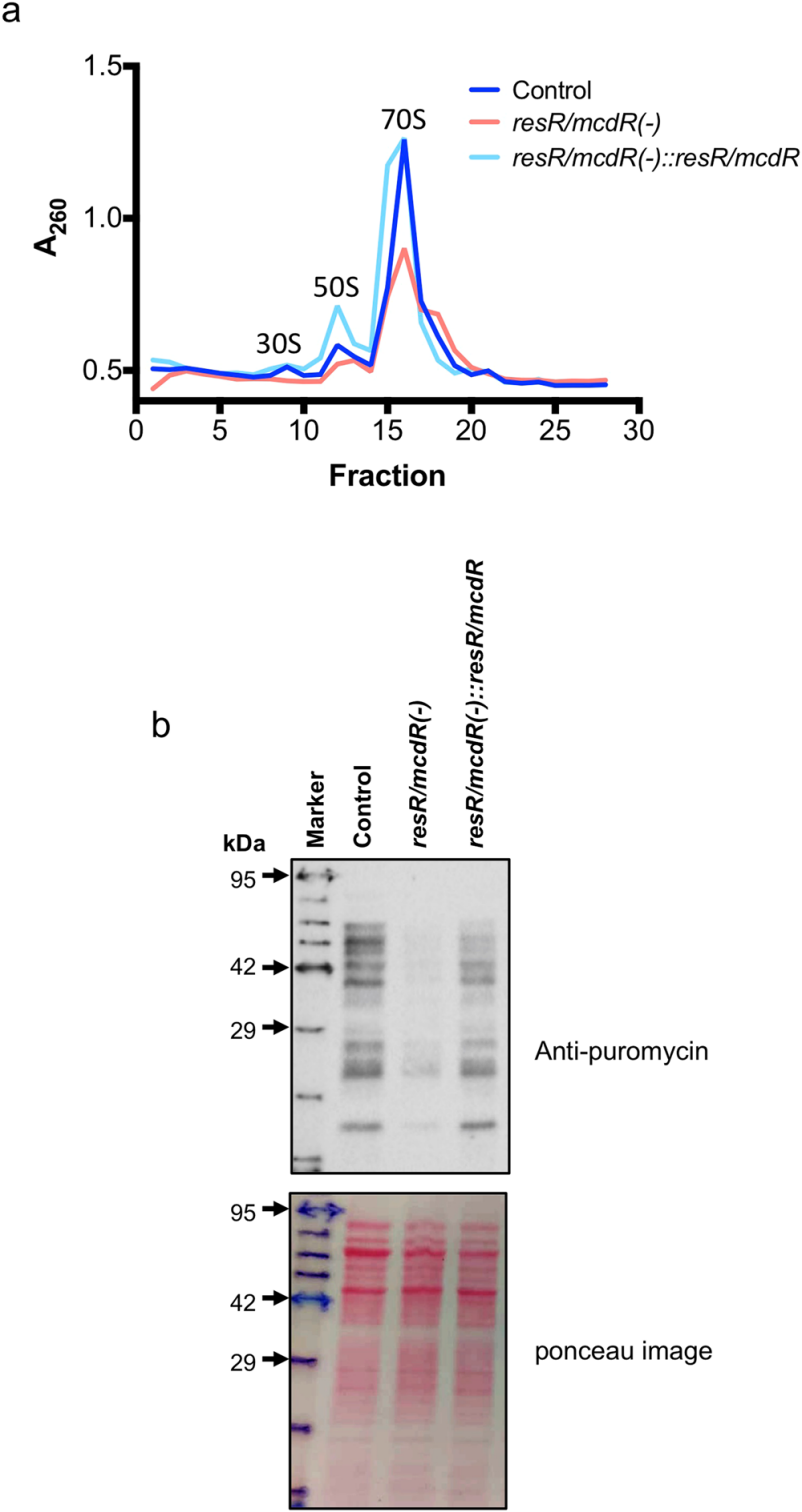
ResR/McdR regulates protein synthesis in Mtb. **a** Ribosome profile of Mtb strains. Different subunits of ribosome were fractionated by ultracentrifugation of equal amount of lysates from empty vector control, *resR/mcdR(−)* and *resR/mcdR(−)::resR/mcdR* strains of Mtb mc^2^ 7902, after 7 days of incubation with 50 ng/ml ATc. Values of absorbance at 260 nm (A_260_) from a total of 30 fractions were plotted in a graph showing the status of 70 S ribosome as well as small (30 S) and large (50 S) subunits in all the three samples. **b** Effect of *resR/mcdR* silencing on initiation of protein synthesis in Mtb. Empty vector control, *resR/mcdR(−)* and *resR/mcdR(−)::resR/mcdR* strains of Mtb Erdman were incubated with 50 µg/ml puromycin for 1 hour after 7 days of ATc treatment. Lysates prepared from the respective strains were subjected to anti-puromycin immunoblotting, which reveals significant inhibition of newly translated proteins upon *resR/mcdR* silencing, which is restored by complementation with the wild-type copy of *resR/mcdR*. Immunoblotting was performed with an equal amount (30 µg) of protein lysates, as ascertained by the ponceau S staining of the membrane. The arrows on the left in **b** indicate the positions of molecular weight markers. Molecular weight markers were accentuated by hand as the signal faded after several washes of the blot. kDa, kilo Dalton.

Since the majority of ribosomal genes are downregulated in *resR/mcdR(−)*, we sought to determine whether the downregulation of *resR/mcdR* indeed affects the total amount of ribosomes available for translation. Ribosome profile of control, *resR/mcdR(−)* and *resR/mcdR(−)::resR/mcdR* strains were analyzed by ultracentrifugation, as described^26^. The results reveal a ~50% reduction in the overall yield of translating ribosome upon depletion of *resR/mcdR*, which is restored by expressing the wild-type *resR/mcdR* in the knockdown strain (Fig. 5a). Next, we examined the effect of conditional knockdown of *resR/mcdR* on the global protein synthesis in Mtb Erdman. All three strains, viz., control, *resR/mcdR(−)* and *resR/mcdR(−)*::*resR/mcdR* were subjected to surface sensing of translation (SUnSET), as described in the Methods. The SUnSET assay involves the incorporation of an amino-nucleoside antibiotic puromycin in the elongating polypeptide chain followed by immunoblotting of lysate with anti-puromycin antibodies^27^. As presented in Fig. 5b, one hour of incubation of these strains with 50 µg/ml puromycin yields intense signals with control and *resR/mcdR(−)*::*resR/mcdR* complemented lysates but not with those prepared from *resR/mcdR(−)* knockdown strain (Fig. 5b and Supplementary Fig. 10). Taken together, these results show that ResR/McdR is vital for maintaining protein translation in Mtb.

### ResR/McdR regulates the expression of the *rplN* operon

**Fig. 6:**
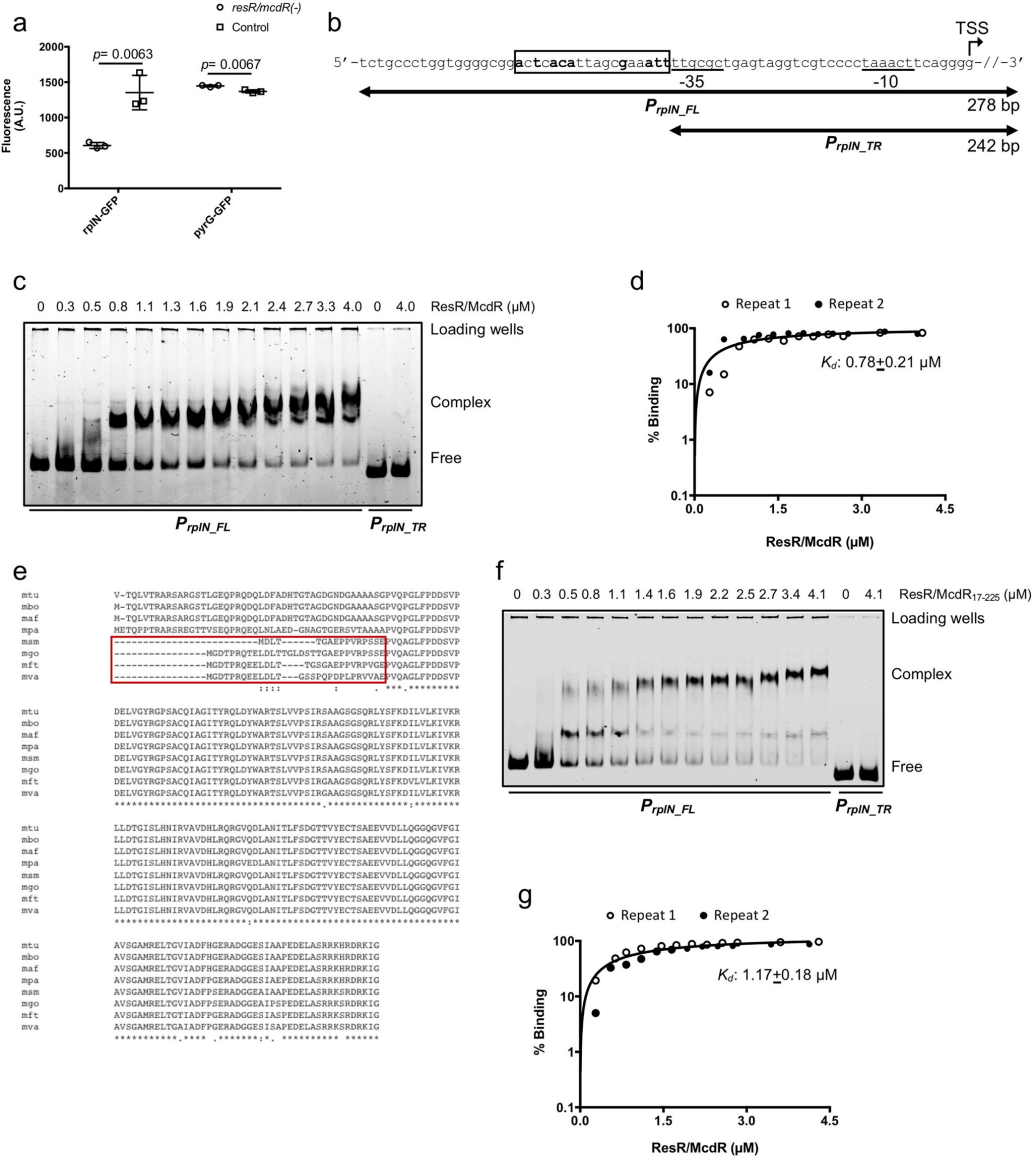
Recognition of *P*_*rplN*_ by ResR/McdR. **a** Analysis of promoter activity by GFP reporter assay. Effect of *resR/mcdR* silencing on activity of *P*_*rplN*_ was estimated by using GFP reporter assay. Estimation of GFP fluorescence reveals ~3-fold reduction under regulation of *P*_*rplN*_ (rplN-GFP) but not under a control promoter, *P*_*pyrG*_ (pyrG-GFP) upon *resR/mcdR* silencing (circle) compared to control (square). **b** Analysis of *P*_*rplN*_ sequence used in EMSA. The TSS site is marked by bent arrow and the underlined sequences represent −35 and −10 promoter elements. Base positions in the respective *P*_*rplN*_ derivatives are shown by double-headed arrows. The putative ResR/McdR-recognition sequence in the *P*_*rplN_FL*_ is shown in black box. The conserved residues are highlighted in bold-face type. **c** Analysis of ResR/McdR binding with *P*_*rplN*_ by EMSA. Binding was performed by using different concentrations of ResR/McdR with *P*_*rplN_FL*_, which reveals ResR/McdR dose-dependent complex formation with the full-length promoter. Notably, absence of complex with *P*_*rplN_TR*_ confirms the sequence-specific binding of ResR/McdR with the proposed recognition sequence in *P*_*rplN_FL*_ promoter. **d** Analysis of ResR/McdR binding kinetics with *P*_*rplN_FL*_. The graph shows the percentage of total DNA probe forming complex at the respective concentrations of ResR/McdR, as shown in **c**. **e** Multiple sequence alignment of ResR/McdR derivatives. Alignment of ResR/McdR homologs from the slow-growing Mtb complex bacteria (mtu, *M. tuberculosis*; mbo, *M. bovis*; maf, *M. africanum*; mpa, *M. avium subspecies paratuberculosis*) and the fast-growing mycobacteria (msm, *M. smegmatis*; mft, *M. fortuitum*; mva, *M. vanbaalenii*) reveals a high level of variability near the N-terminus, which is shown in a red box. **f** Dose-dependent binding of ResR/McdR_17-225_ with *P*_*rplN*_. Binding was analyzed by EMSA, typically as described above in **c**. **g** Assessment of ResR/McdR_17-225_ binding kinetics with *P*_*rplN_FL*_. The graph shows the percentage of total DNA probes forming a complex at the respective concentrations of ResR/McdR_17-225_, as shown in **f**. The dissociation constant (*K*_*d*_) was determined by using GraphPad Prism v7.0e software. Data represent mean ± s.d. (shown by error bars) of multiple (*n* = 3) biological repeats in **a**. Non-linear fit of data from multiple (*n* = 2) biological repeats are shown in **d**, **g**. *p* values in **a** were obtained for the respective samples after comparison with control, as described in Methods.

In a recent study, a consensus DNA sequence recognized by ResR/McdR was reported. It was found that this regulator prefers an 18 bp reverse complementary sequence 5’-AATnACA-nnnn-TGTnATT-3’ present in the 5’-UTR of a few *M. smegmatis* genes such as *MSMEG_1831* (*whiB2*)*, MSMEG_0833* and *MSMEG_5468* with 100% similarity^15^. Akin to the previous study, we also find a strong and sequence-specific binding of Mtb ResR/McdR purified from *E. coli* (Supplementary Fig. 11) with Mtb *whiB2* promoter, which exhibits a dissociation constant (*k*_*d*_) of 0.33 ± 0.15 µM (Supplementary Fig. 12). To gain an insight into Mtb genes that might be directly regulated by ResR/McdR, we thoroughly analyzed the upstream region of ResR/McdR regulons which revealed 113 sequences from 100 genes containing the potential ResR/McdR-recognition motifs (Supplementary Data 3). One of these sequences belongs to *rplN* which encodes for a 50 S ribosomal protein L14. Since protein translation is majorly impacted by the downregulation of *resR/mcdR* in Mtb, we selected the representative promoter region of *rplN* for further investigation of its regulation by ResR/McdR. Analysis of the *rplN* locus in Mtb indicates that it is transcribed in an operon^28^ with nine genes downstream to it, namely *rplX, rplE, rpsN1, rpsH, rplF, rplR, rpsE, rpmD* and *rplO* that encode for ribosomal proteins (Supplementary Fig. 13). Importantly, the expression of these genes is consistently downregulated across multiple biological replicates (Fig. 4a). To test if the altered expression of *rplN* operon in *resR/mcdR(−)* indeed involves ResR/McdR-mediated regulation of its promoter activity, the 5’-UTR of *rplN* was cloned upstream to green fluorescent protein-encoding gene (*gfp*), as described in the Methods. The resulting plasmid, pPro_rplN_-gfp was introduced in both the empty vector control and the *resR/mcdR(−)* knockdown strains for estimation of GFP fluorescence. As a control, the expression of *gfp* was also analyzed in these strains under the regulation of the promoter of an unrelated gene *pyrG* (annotated as *P*_*pyrG*_), which didn’t show any change in the RNASeq experiment. Figure 6a demonstrates that the *gfp* expression driven by *P*_*rplN*_, but not by the *P*_*pyrG*_, is reduced upon *resR/mcdR* silencing by nearly threefold (*p* < 0.05), which indicates a specific effect of ResR/McdR on *rplN* promoter activity (Fig. 6a and Supplementary Data 1).

Subsequently, the *rplN* promoter fragment (annotated as *P*_*rplN_FL*_) was PCR amplified and subjected to the electrophoretic mobility shift assay (EMSA) using purified ResR/McdR. The *P*_*rplN_FL*_ exhibits ResR/McdR-recognition sequence with 3 mismatches (5’-AcTcACA-ttag-cGaaATT-3’) immediately upstream to the −35 element (Fig. 6b and Supplementary Data 3). Simultaneously, a truncated version of *P*_*rplN_FL*_ lacking the potential ResR/McdR binding sequence (termed as *P*_*rplN_TR*_), was used as control (Fig. 6b). The EMSA results show that ResR/McdR strongly binds with *P*_*rplN_FL*_ in a dose-dependent manner with a dissociation constant (*K*_*d*_) of 0.78 ± 0.21 µM (Fig. 6c, d and Supplementary Data 1). Importantly, no complex formation is seen between ResR/McdR and *P*_*rplN_TR*_ even with the maximum concentration of 4.0 µM (Fig. 6c). Overall, our results confirm the direct regulation of ribosomal genes by ResR/McdR, which further corroborate the crucial role of this essential TR in the maintenance of the protein translation machinery in Mtb.

### The N-terminal disordered region of ResR/McdR is essential for its DNA-binding activity

To gain a mechanistic insight into the regulation of genes by ResR/McdR, we sought to identify the critical domain(s) in ResR/McdR which is important for its DNA-binding activity. As mentioned, ResR/McdR comprises highly disordered terminal sequences (Supplementary Fig. 3a). Interestingly, the N-terminal sequence of Mtb ResR/McdR, which is highly conserved across Mtb complex bacteria, shows a high level of variation in its counterparts from the fast-growing mycobacteria (Fig. 6e). This is particularly intriguing because the rest of the protein sequences exhibit >95% conservation. EMSA results reveal that the affinity of purified ResR/McdR^MS^ towards *P*_*rplN_FL*_ is relatively lower (*K*_*d*_ = 1.4 ± 0.24 µM) in comparison to its counterpart from the slow-growing *M. tuberculosis* (Supplementary Fig. 14). These findings indicate that the N-terminal region of the Mtb ResR/McdR may be crucial for the DNA-binding activity. To further investigate, we expressed and purified truncated versions of ResR/McdR with short (16 amino acids) and long (68 amino acids) deletions at the N-terminus (annotated as ResR/McdR_17-225_ and ResR/McdR_69-225_, respectively), as described in the Methods. Since we were unable to obtain the truncated proteins with the 6x histidine tag in the soluble fraction, all the proteins were purified with the N-terminal GST tag (Supplementary Fig. 11a). Unfortunately, the removal of 68 amino acid residues from the N-terminus adversely affects purity as well as the secondary structure of the purified ResR/McdR with the complete loss of the α-helix, as assessed by the CD spectroscopy. In contrast, ResR/McdR_17-225_ is purified to homogeneity maintaining its secondary structure conformation (Supplementary Figs. 11a–c). Subsequently, the binding kinetics of the purified GST-ResR/McdR_17-225_ with the *P*_*rplN_FL*_ was performed by EMSA, which shows a reduction in its DNA-binding activity. The ResR/McdR_17-225_ dose-dependent kinetics reveals that it binds the *P*_*rplN_FL*_ promoter in a sequence-specific manner with a *K*_*d*_ of 1.17 ± 0.18 µM (Fig. 6f, g and Supplementary Data 1). These results further corroborate the essential requirement of the distinct disordered N-terminal sequence in the ResR/McdR for promoter recognition.

### Depletion of *resR/mcdR* decreases Mtb resilience to antibiotics

**Fig. 7:**
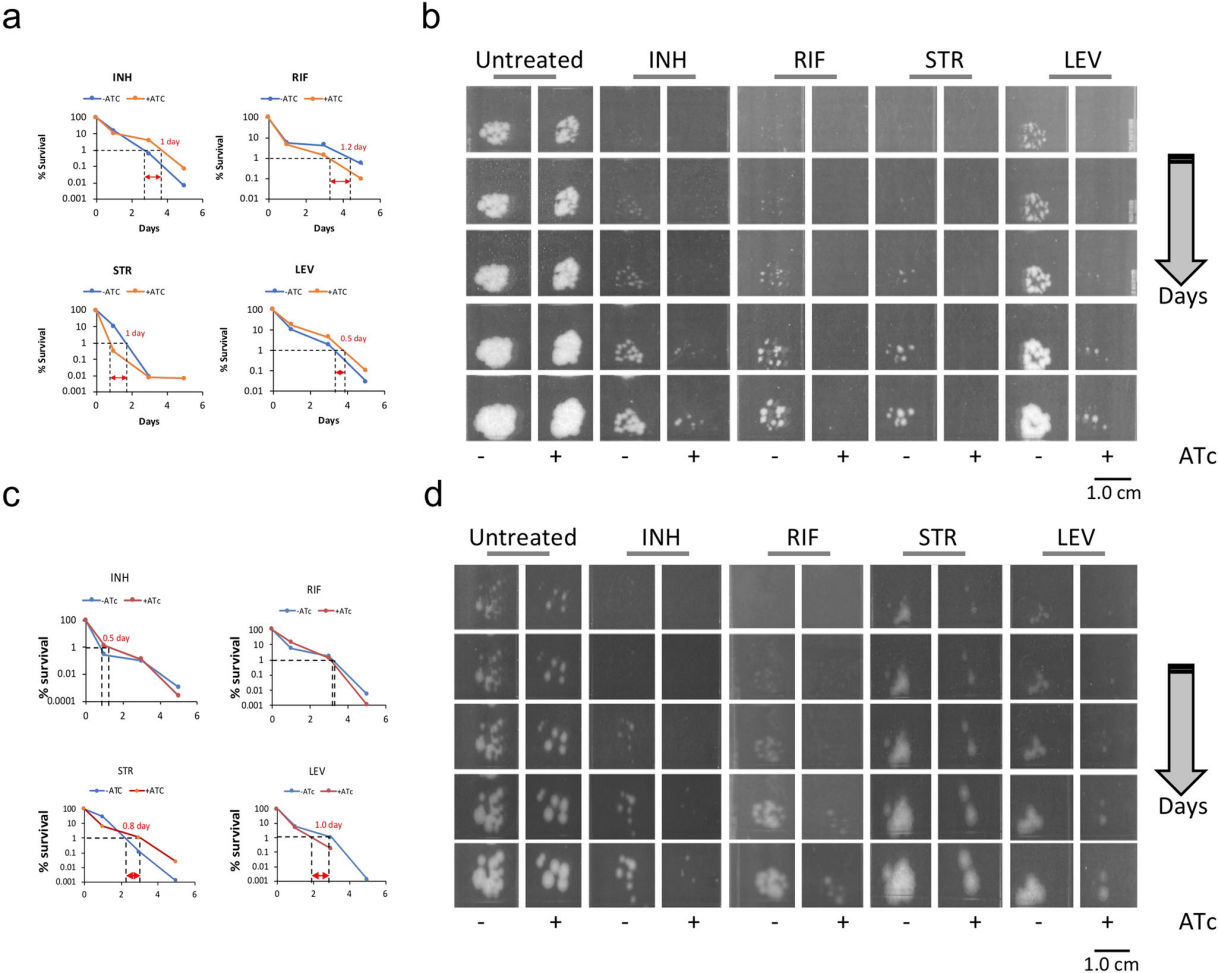
Effect of suppression of *resR/mcdR* and *rplN* on post-antibiotic recovery of Mtb. **a** Time-kill kinetics of the *resR/mcdR(−)*. Time-kill kinetics was examined in response to treatment with isoniazid (INH), rifampicin (RIF), streptomycin (STR), and levofloxacin (LEV). The MDK_99_ by each drug, after exposure to 10× MIC is depicted by the dashed line. **b** Post-antibiotic recovery dynamics of *resR/mcdR(−)*. Representative images, captured between 13 and 21 days of incubation, depict the post-antibiotic recovery dynamics of Mtb depleted with *resR/mcdR*. **c** Time-kill kinetics of the *rplN(–)*. Time-kill kinetics was examined in response to treatment with 10× MIC of different drugs, as mentioned in **a**. The MDK_99_ by each drug is depicted by the dashed line. **d** Post-antibiotic recovery dynamics of *rplN(–)*. Representative images, captured between 13 and 21 days of incubation, depict the post-antibiotic recovery dynamics of Mtb depleted with *rplN*. Scale bars in **b**, **d** are shown for size reference.

As hitherto mentioned, certain clinical isolates of Mtb with point mutations in ResR/McdR can survive antibiotics in TB patients, and the Mtb H37Rv strains with the similar mutations exhibit faster recovery, post-antibiotic treatment^16^. To understand the impact of *resR/mcdR* depletion on PAE, we determined the time of recovery of ATc-treated and untreated *resR/mcdR(−)* strain following 24 hours of exposure to different drugs, as mentioned in the Methods. The time-kill assay using a panel of four antibiotics, including two first-line (rifampicin and isoniazid) and two second-line (levofloxacin and streptomycin) drugs, reveals no dramatic change in the susceptibility of the *resR/mcdR(−)* to any of these antibiotics (Fig. 7a and Supplementary Data 1). The minimum duration of time required to kill both ATc-treated and untreated bacteria by 99% (MDK_99_) for these drugs differ marginally by ~0.5–1 day (Fig. 7a). In contrast, the *resR/mcdR(−)* treated with ATc exhibits a significant delay in the appearance of colonies following treatment with these drugs in comparison to the ATc-untreated bacteria (Fig. 7b). Notably, we do not find any change either in the kill kinetics or in the PAE of the control strain (Supplementary Fig. 15a, b), indicating a specific effect of *resR/mcdR* knockdown on the PAE.

### ResR/McdR-regulated expression of *rplN* is critical for Mtb drug resilience

Although the underlying cause of PAE is not understood well, there are a few reports indicating that mRNA transcription, protein translation, and efflux machinery affect the PAE^29–32^. Interestingly, bacterial resilience to drugs decreases with a reduction in the number of effective ribosomes. Bacteria with fewer *rrn* operons take longer time to revive following the drug treatment, than those with more *rrn* operons^29^. Since ResR/McdR regulates several genes including *whiB2* and *rplN*, we sought to determine if ResR/McdR-mediated PAE involves any of these genes. Knockdown strains of *whiB2* and *rplN* were created by using CRISPRi. Briefly, *whiB2(–),* and *rplN(–)* strains were cultured in 7H9-OADS medium at OD_600_ of ~0.10 followed by incubation with 25- and 50 ng/ml ATc for 4 days to obtain suppression of these genes without any adverse effect on growth compared to the respective ATc-untreated controls. Bacterial kill kinetics and the post-antibiotic recovery were subsequently observed for both strains, typically as described above. Our results show that MDK_99_ for all four drugs is not greatly affected upon silencing of the respective genes (Fig. 7c, Supplementary Fig. 15c and Supplementary Data 1). Interestingly, the recovery time of ATc-treated *rplN(–)* is significantly increased following drug treatment relative to ATc-untreated bacteria (Fig. 7d). Contrary to this, suppression of *whiB2* does not show any change in mycobacterial resilience to these drugs (Supplementary Fig. 15d).

## Discussion

Antimicrobial resistance in bacterial pathogens including Mtb is a global concern^33^. While genetic mutations are typically associated with drug resistance, it is worth noting that a subpopulation of bacteria can temporarily develop resistance without undergoing mutation^34^. Though brief exposure to antibiotics often results in prolonged suppression of growth, bacteria have evolved a marvelous capacity to recover from the toxic effects of drugs by altering their physiology leading to antibiotic resilience. The post-antibiotic effect is considered an important parameter for the design of treatment regimens across many bacterial pathogens. It is affected by various factors such as the concentration of the antibiotic, the dosage of the target protein, the vulnerability of the drug target, and the residence time of the antibiotic to occupy the target and inhibit its activity^35^. Alteration of the ribosomal content in the cell or intra-population variability in cell-division and death rates during and after antibiotic treatment also controls post-antibiotic recovery in other microorganisms^29,36,37^. At the genetic level, PAE is influenced by perturbation of mRNA transcription^31,32^, protein synthesis^29,30^, and efflux machinery^30^. Recently, ResR/McdR has been implicated in attributing drug resilience in the TB pathogen, however, it remains to understand how this essential transcription regulator controls drug resilience and what is the underlying cause of its essentiality in Mtb.

The current study is performed to comprehend the function of the ResR/McdR regulator in the pathogenic Mtb Erdman. Our results establish that *resR/mcdR* is independently transcribed with the help of a promoter element comprising SigA-recognition motifs^23^ 5’-TACTTT-3’ and 5’-GTGCCT-3’ at the −10 and −35 positions, respectively (Fig. 1a). SigA is a housekeeping sigma factor that is essential for Mtb growth. The presence of SigA-recognition motifs in the *resR/mcdR* promoter further explains the constitutive expression of ResR/McdR and reiterates its critical requirement in Mtb, as also evidenced by the poor extracellular proliferation of bacteria depleted with *resR/mcdR*. Noteworthy to mention, bacteria proliferate at a relatively slower rate compared to control during the initial period of silencing of *resR/mcdR*, and the growth becomes static at later time points (Fig. 1c). Importantly, the massive killing of *resR/mcdR* mutant in mice organelles (Fig. 2d, e) highlights its essential requirement in Mtb to withstand the host assault.

Global transcriptional profiling of *resR/mcdR(–)* by RNA sequencing sheds important light on the role of ResR/McdR in Mtb. To avoid any growth-related pleiotropic effect on gene expression profile, conditions for silencing of *resR/mcdR* were carefully selected such that the growth of control and *resR/mcdR(−)* remains comparable. Although complete suppression of *resR/mcdR* could not be obtained under these conditions, yet the expression of nearly 1/5^th^ of Mtb genes is altered, which indicates a massive reprogramming of the mycobacterial transcriptional machinery (Fig. 3). In addition to other genes, the expression of several TRs is also affected in the *resR/mcdR(–)* strain which might indirectly contribute to the modulation of such a vast number of transcripts. For instance, many differentially expressed genes exhibit recognition motifs in their respective promoter regions for SigA, which is downregulated by ~50% upon depletion of *resR/mcdR* (Supplementary Data 2). Since these results were obtained after 4 days of treatment with ATc, it will be interesting to examine whether similar changes in the gene expression profile are obtained upon immediate loss of *resR/mcdR* using alternate strategies such as the dual inducible system^38^.

Perturbation of a large number of metabolic genes also disturbs the cellular metabolic profile of the *resR/mcdR(−)* strain of Mtb. Downregulation of ribosomal genes as well as translating ribosomes and subsequent protein synthesis in the *resR/mcdR(−)* provides evidence for a possible role of ResR/McdR in bacterial protein translation machinery (Figs. 4 and 5). Abnormal protein synthesis in the *resR/mcdR*-depleted strain is further corroborated by the accumulation of several tRNAs and amino acids, likely due to their underutilization. Regulation of protein synthesis by ResR/McdR further explains its essential requirement for bacterial growth. Several studies demonstrate a direct relation between the rates of bacterial growth and the number of ribosomes in a cell^39,40^. Indeed the bacterial growth rate in the nutrient-rich environment is affected by the average number of ribosomes contributing to protein translation^29^. Notably, several of the ResR/McdR regulons, including *rplN* operon genes, exhibit the presence of potential ResR/McdR-recognition motifs in their 5’-UTRs, indicating direct regulation. Indeed we show that *rplN* promoter is under direct control of ResR/McdR (Fig. 6a). Notably, ResR/McdR can recognize a specific DNA sequence motif in *P*_*rplN*_, which marginally deviates from the known consensus present in a select set of promoters including *P*_*whiB2*_, albeit with relatively compromised affinity (Fig. 6c, d). Together, these results indicate considerable flexibility in sequence preference by Mtb ResR/McdR, which seems to tolerate a few mismatches in its recognition sequence. The presence of similar sequences in the 5’-UTR of some of the ResR/McdR regulons that are involved in mRNA transcription, lipid biosynthesis, and respiration, further explains widespread gene expression changes upon *resR/mcdR* silencing.

Although ResR/McdR is present across different actinomycetes families of bacteria, sequence conservation is restricted primarily to the central region comprising the HTH motif, whereas the adjacent sequences at the N- and C-termini are highly variable (Fig. 6e). As described above, the terminal sequences comprise disorder-promoting residues and do not exhibit similarity with known motifs. Sequence variability in the N-terminal region of ResR/McdR among different mycobacterial species intrigued us to examine the role of the terminal disordered regions in the activity of the Mtb ResR/McdR. Our results show that the N-terminal region is critical for DNA recognition (Fig. 6f, g). The compromised DNA-binding activity of *M. smegmatis* protein towards *P*_*rplN*_ relative to its Mtb counterpart further reiterates the importance of the N-terminal sequence in the transcriptional regulation by ResR/McdR (Supplementary Fig. 14). The importance of the intrinsic disordered regions (IDRs) outside the DNA-binding domains of the TRs has been shown recently in the *Saccharomyces cerevisiae* strain BY4741, wherein it was reported that the long IDRs in the Msn2 and Nrg2 TRs are crucial for their localization to the target promoters^41^. The IDR-directed binding in these regulators is not domain-specific, rather it involves several weak sensing determinants scattered throughout the IDR sequence which accelerate the detection of core binding region by rapidly localizing TRs to broad DNA regions around these sites fielded^41^. A follow-up study is required to better understand how the disordered N-terminal sequence regulates the DNA-binding activity of Mtb ResR/McdR.

As anticipated, we find prolonged PAE in *resR/mcdR*-silenced bacteria which take longer duration to recover from the effect of antibiotic treatment as against ATc-untreated control. Notably, the susceptibility trend is similar to that observed with the knockdown of *rplN* but not with *whiB2*, which exhibits a similar profile obtained with the control strain. Various studies highlight the regulation of PAE by the availability of effective ribosomes. A gradual decrease in ribosome content in the cell leads to a concomitant increase in the recovery duration of bacteria post-antibiotic treatment, as bacteria take time to replenish the functional ribosomes and replicate^29,30^. These observations and our findings together provide mechanistic insights into the regulation of PAE in the TB pathogen. ResR/McdR-dependent control of *rplN* expression and subsequent protein synthesis machinery in Mtb seems pivotal for attributing resilience to various antibiotics. It will be riveting to examine the impact of point mutations in ResR/McdR that are enriched in clinical isolates of Mtb on the bacterial transcription and translation machinery. We further propose screening of small molecule inhibitors against Mtb ResR/McdR that can be used as adjunctive drugs with standard therapy to shorten the TB treatment in humans.

## Methods

### Bacterial strains and culture conditions

*Escherichia coli* strain DH5α (Thermo Fisher) was used for the propagation of plasmids, whereas *E. coli* BL21 DE3 (Novagen) was used for the expression and purification of ResR/McdR protein. Mtb Erdman was obtained from Dr. Ramandeep Singh at THSTI, India, and Mtb H_37_Rv mc^2^ 7902^42^ strain was obtained from Dr. William Jacobs at Albert Einstein College of Medicine, NY, USA. While *E. coli* was grown in the LB medium (Becton Dickinson), Mtb was cultured in Middlebrook 7H9 containing 0.05% tyloxapol (Merck) or Middlebrook 7H11 without detergent. Both these media were supplemented with 1× OADS (oleic acid-albumin-dextrose-saline) and 0.5% glycerol. Liquid cultures were grown either in 50 ml tubes or in flasks containing not more than the one-third volume of bacterial cultures, whereas plates were incubated at 37 °C. We used 50 µg/ml kanamycin, 50 µg/ml zeocin, 50 µg/ml ampicillin, and 150 µg/ml hygromycin for *E. coli* whereas for Mtb 25 µg/ml kanamycin, 25 µg/ml zeocin, and 50 µg/ml hygromycin were used, wherever needed.

### Construction of recombinant strains of Mtb

Conditional silencing of *resR/mcdR*, *rplN*, and *whiB2* in Mtb was achieved by CRIPSRi, typically as described earlier^43^. Briefly, a set of oligonucleotides resR/mcdR_Cr-UP and resR/mcdR_Cr-DN (Supplementary Data 4), specific to the 5’-UTR of *resR/mcdR* were annealed and cloned adjacent to the Cas9 handle sequence at the *Afl II-Acl I* sites under the regulation of the TetR-dependent P_myc1tetO_ promoter in a Kan^R^ integrative plasmid pDcas9, a derivative of pTetInt-*dcas9*^43^. The resulting plasmid, annotated as pDcas9-*resR/mcdR*, was verified by DNA sequencing. For conditional suppression of *rplN* and *whiB2*, oligonucleotides rplN_Cr-UP – rplN_Cr-DN and whiB2_Cr-UP – whiB2_Cr-DN (Supplementary Data 4) were annealed and cloned in pGrna vector as earlier^43^. The recombinant Hyg^R^ plasmids, pGrna-*rplN* and pGrna-*whiB2* were verified by DNA sequencing.

Both the pDcas9 and pDcas9-*resR/mcdR* were electroporated in Mtb Erdman as well as in Mtb mc^2^ 7902 and the resulting Kan^R^ strains were annotated as control and *resR/mcdR(−)*, respectively. The empty pGrna plasmid as well as pGrna-*rplN* and pGrna-*whiB2* were transformed in Kan^R^ dCas9-expressing Mtb mc^2^ 7902 cells and the resulting Kan^R^-Hyg^R^ strains were treated as control, *rplN(−)* and *whiB2(−)*, respectively. Silencing of *resR/mcdR* and *rplN* genes was achieved in the respective knockdown strains by incubating cultures at initial OD_600_ of ~0.10 with 50 ng/ml anhydrotetracycline (ATc) for 4 days unless specified. Suppression of *whiB2* was achieved by incubation of the *whiB2(−)* cultures at an initial OD_600_ of ~0.10 with 25 ng/ml ATc for 4 days.

Effect of depletion of *resR/mcdR* in the *resR/mcdR(−)* strain was restored by complementation with the wild-type copy of the gene. Briefly, the *resR/mcdR* ORF was PCR amplified from the Mtb genomic DNA with the help of gene-specific primers resR/mcdR-F and resR/mcdR-R containing *Nde I and Hind III* overhangs, respectively, (Supplementary Data 4). The resulting PCR amplicon was restriction digested with *Nde I and Hind III* enzymes and cloned at the same sites in a Hyg^R^ replicative plasmid, pTetR^44^ under the regulation of P_myc1tetO_ promoter, yielding a recombinant plasmid named pTetR-*resR/mcdR*. The sequence of *resR/mcdR* in pTetR-*resR/mcdR* was verified by DNA sequencing. Subsequently, the pTetR-*resR/mcdR* plasmid was electroporated in the *resR/mcdR(−)* and Kan^R^-Hyg^R^ colonies of *resR/mcdR(−)*::*resR/mcdR* complemented strain was verified by colony-PCR.

### Infection of animals

Both the control and the *resR/mcdR(−)* strains of Mtb Erdman were cultured in the 7H9-OADS broth medium to exponential phase, and washed with 1× phosphate-buffered saline (PBS) followed by multiple passaging through a syringe to disperse clumps. Infection of 8–9 week old female BALB/c mice (bred at the Small Animal Facility at Translational Health Science and Technology Institute (THSTI)) was performed through aerosol route by using ~5 × 10^7^ CFU/ml of each of these strains using Glas-Col Inhalation Exposure System, which resulted in the infection of ~300–500 bacteria per animal, as estimated by lung CFU counts on the day 1 post-infection. After 3 weeks of infection, groups of infected mice received 1.5 mg/ml doxycycline (Merck) in drinking water containing 5% sucrose twice a week, whereas the control groups received 5% sucrose. Bacterial burden was determined at regular intervals from both the doxycycline-treated and untreated groups by plating serial dilutions of lung and spleen homogenates onto 7H11 agar plates containing carbenicillin (0.1 µg/ml), polymyxin B (0.30 µg/ml), cycloheximide (0.9 µg/ml), trimethoprim (22 ng/ml) and kanamycin (25 µg/ml). Plates were incubated at 37 °C and colonies were counted after 4 weeks. Animal infection experiment was conducted after due approval from the Institutional Biosafety Committee (IBSC Reference No. 382, dated 28-12-2021) and the Institutional Animal Ethics Committee (Approval No. IAEC/THSTI/77, dated 29-08-2019) of THSTI.

### Cloning, expression, and purification of ResR/McdR

To obtain the purified full-length ResR/McdR (ResR_1-225_), the *resR/mcdR* ORF was first obtained from pTetR-*resR/mcdR* by restriction digestion with *Nde I and Hind III*, which was subsequently cloned at the same sites either in pET28 (Novagen) for expression as N-terminal 6x His-tagged protein or in a derivative of *E. coli* expression vector pGEX-6P-1 (Merck) for its expression with GST tag at the N-terminus. The truncated versions of *resR/mcdR* viz., *resR/mcdR*_*17-225*_ and *resR/mcdR*_*69-225*_ were amplified using the pGEX-ResR/McdR_1–225_ as template with the help of resR/mcdR_17_-F – resR/mcdR-R and resR/mcdR_69_-F – resR/mcdR-R primer pairs, respectively (Supplementary Data 4). The PCR amplicons were subjected to restriction digestion with *Nde I and Hind III* enzymes and cloned at the same sites in the pET28 as well as modified pGEX-6P-1 plasmids. The sequence of cloned fragments in the recombinant clones was verified by DNA sequencing.

For protein expression, the respective plasmids were used to transform *E. coli* BL21 DE3, and colonies were obtained on antibiotic-containing LB agar plates after overnight incubation at 37 °C. A single colony was inoculated in the LB broth containing antibiotics and the bacteria were allowed to grow for overnight at 37 °C. Induction of proteins was achieved by treatment of secondary cultures at OD_600_ of 0.6 with 1.0 mM IPTG for 16 h at 18 °C. Bacteria were subsequently pelleted and washed once with the lysis buffer (50 mM Tris-HCl, pH 8.0, 150 mM NaCl and 10% glycerol). Lysis was performed in the lysis buffer containing 1× protease inhibitor cocktail (Merck) with the help of PandaPLUS laboratory homogenizer, followed by centrifugation to remove cell debris.

For purification of the His-tagged proteins, lysates prepared from *E. coli*::pET28-*resR/mcdR* were incubated with Ni-NTA resin (Qiagen), prewashed with the lysis buffer, for 3 hours at 4 ˚C. Unbound proteins were removed by extensive washing of the resin, each with 3× column volumes of lysis buffer containing 20 mM imidazole. Subsequently, the recombinant proteins were eluted after incubation of resin with the lysis buffer containing 200 mM imidazole.

For purification of the GST-tagged proteins, additional sodium chloride was added in the clarified lysates to a final concentration of 1.0 M, along with 5.0 mM β-mercaptoethanol and 1.0 mM of EDTA. Lysates were incubated with glutathione-Sepharose resin for 8 hours at 4 °C to immobilize the GST-tagged proteins on the resin. Unbound proteins were removed by extensively washing the resin, each with 3× column volumes of the wash buffer (lysis buffer containing 0.50 M NaCl) followed by washing with 1× column volume of the lysis buffer containing 1 mM reduced glutathione. Subsequently, the resin was incubated with the elution buffer (lysis buffer containing 10 mM reduced glutathione) and the eluted fractions of GST-tagged ResR/McdR proteins were collected in different tubes.

The purity of eluted fractions of the wild-type and truncated version of ResR/McdR was determined by SDS-PAGE followed by Coomassie Brilliant Blue staining of the gel. Protein fractions exhibiting >90% purity were pooled and dialyzed against the lysis buffer. The purified proteins were stored in multiple aliquots at −80 °C for subsequent use.

### Cloning, expression, and purification of ResR/McdR^MS^

In order to purify the wild-type ResR/McdR^MS^, the *MSMEG_3644* ORF was amplified using genomic DNA of *M. smegmatis* with the help of primer pair MSMEG_3644-F and MSMEG_3644-R (Supplementary Data 4). The PCR amplicon was subjected to restriction digestion with *Nde I* and *Hind III* enzymes and cloned at the same sites in the modified pGEX-6P-1 plasmid to obtain expression of the recombinant protein with GST tag at the N-terminus. The sequence of cloned fragments was verified by DNA sequencing before proceeding for expression and purification of ResR/McdR^MS^, which was performed typically as described above.

### Size exclusion chromatography (SEC)

Conformation of the purified ResR/McdR was analyzed by using analytical Superdex-200 Increase 10/300 GL (Cytiva). An aliquot of purified protein (~100 µg/500 µl) prepared in a buffer containing 100 mM Tris-HCl, pH 8.0, and 150 mM NaCl, was loaded on the column pre-equilibrated with same buffer, and resolved using an AKTA purifier system at a flow rate of 0.4 ml/min. Proteins in the eluted fractions were detected by analyzing the absorbance at 280 nm, and the values were subsequently used to plot the curve. Presence of protein in the eluted fractions was also determined by SDS-PAGE. To assess the molecular mass of the protein, a standard curve was prepared using known size-exclusion chromatography standards (Merck) after determining the void volume.

### Circular dichroism spectroscopy

To determine the secondary structure content in the purified proteins, CD spectra were obtained using JASCO J-815 spectropolarimeter (Jasco). Briefly, proteins were dialyzed and diluted in 10 mM phosphate buffer (pH 7.40) to adjust the final concentration of 200 µg/ml. Spectra were obtained with 300 µl sample in quartz cuvette of 2.0 mm path length using a range of wavelength from 190 to 300 nm, at 18 °C with a data point interval of 1.0 nm. An average of five CD measurements was taken after subtracting the values obtained with buffer alone, which were subsequently plotted to obtain the CD curve for each protein.

### Extraction of RNA and cDNA synthesis

Total RNA was extracted by bead-beating lysis of bacteria in Trizol reagent, as instructed by the manufacturer (Thermo Fisher). RNA samples were treated with DNA-free DNase I (Ambion), and cDNA was synthesized using superscript III reverse transcriptase, according to the manufacturer’s instructions (Thermo Fisher).

### Determining the TSS of the *resR/mcdR* mRNA

The transcription start site (TSS) of the *resR/mcdR* mRNA was determined by the 5’-RACE (rapid amplification of cDNA ends) technique, as described previously^22,45^. Briefly, the DNase-treated RNA was reverse transcribed using resR/mcdR_RT-R primer (Supplementary Data 4), followed by 30 minutes of incubation at 37 °C with RNase H and RNase A. The cDNA was subsequently purified using reaction clean up kit (Qiagen), and subjected to dC tailing with the help of dCTP and terminal transferase (NEB). The dC-tailed cDNA was PCR amplified using Abridged Anchor (Thermo Fisher) and resR/mcdR_RT-R primer pair (Supplementary Data 4). The nested PCR was performed using Abridged Anchor and resR/mcdR_CrUP primer pair (Supplementary Data 4). The PCR products were subsequently sequenced using resR/mcdR_CrUP.

### Quantitative reverse transcription real-time PCR (qRT-PCR)

The qRT-PCR was performed using 50 ng cDNA, gene-specific primers (Supplementary Data 4), and SYBR Green PCR master Mix, as suggested by the manufacturer (Applied Biosystems). Real-time monitoring and quantification were carried out using ABI 7500 Fast Real-Time PCR System (Applied Biosystems), as described previously^46^. Briefly, initial denaturation was performed at 95 ˚C for 10 minutes, which was followed by 40 cycles of amplification involving denaturation at 95 ˚C for 15 seconds and amplification at 60 ˚C for 1 minute. Fluorescence was recorded during each amplification event and cutoff (Ct) values were determined at the end of the reaction by the in-built software. The Ct values were subsequently used for calculation of the fold-change in expression as presented in Equation 1 below:

1 $$	{{{{{\mathrm{Fold}}}}}}{\mbox{-}}{{{{{\mathrm{change}}}}}}\;{{{{{\mathrm{of}}}}}} \;{{{{{\mathrm{ gene}}}}}}\;{{{{{\mathrm{ expression}}}}}}\;{{{{{\mathrm{ in}}}}}} \;{{{{{\mathrm{the}}}}}}\;{{{{{\mathrm{ test}}}}}}\;{{{{{\mathrm{ sample}}}}}} \;{{{{{\mathrm{with}}}}}} \;{{{{{\mathrm{respect}}}}}}\;{{{{{\mathrm{ to}}}}}} \;{{{{{\mathrm{control}}}}}}\\ 	\quad= 2^{\wedge}\left(\;{{{{{\mathrm{Ct}}}}}}^{{{{{{\mathrm{CONTROL}}}}}}}-{{{{{\mathrm{Ct}}}}}}^{{{{{{\mathrm{TEST}}}}}}}\right).$$

### Preparation of Mtb lysates

Whole cell lysates of Mtb were prepared by bead-beating lysis of bacteria in the lysis buffer consisting of 1× protease inhibitor cocktail (Merck) in 1× PBS, followed by centrifugation at 12,000 × *g* for 10 minutes at 4 °C to remove the cell debris. The clarified lysate was passed through 0.22 µM syringe filter, and stored at −80 °C.

### Immunoblotting

Immunoblotting was performed with anti-ResR/McdR antibodies that were commercially raised in rabbits using ResR/McdR-specific immunogenic peptides (ELASRRKHRDRKIG) (Genscript). Whole cell lysates were resolved by denaturing SDS-PAGE using 10% gel and transferred to nitrocellulose membrane by semi-dry electro-transfer method. The membrane was blocked with 5% non-fat dried milk (Bio-Rad) in 1× PBS containing 0.05% tween-20 (PBST) for 1 hour at 37 °C, followed by incubation with anti-ResR/McdR IgGs (0.2 µg/ml in the blocking buffer) for overnight at 4 °C. The membrane was washed thrice with 1× PBS containing 0.1% tween-20 followed by 1 hour of incubation with horseradish peroxidase-conjugated IgG (Cell Signaling Technology) in PBST. Extensive washing of the membrane was subsequently performed with PBST to remove unbound IgGs. Chemiluminescence signals were obtained after incubation of the blot with the Super Signal West Femto (Thermo Fisher) using a gel documentation system (Bio-Rad).

### Whole genome transcriptional analysis by RNA sequencing

Total RNAs were extracted from three biological replicates of Mtb::pDcas9 (control) and Mtb::pDcas9*-resR/mcdR* (*resR/mcdR(−)*) after 4 days of treatment with 50 ng/ml ATc, followed by DNase I treatment. The DNase-treated RNA samples were provided to Clevergene (https://clevergene.in/) for further processing and sequencing. Libraries were prepared according to Illumina’s instructions by Clevergene. The sequence data were generated using Illumina HiSeq, and the quality of data was examined by using FastQC^47^ and MultiQC^48^ software. The data was thoroughly evaluated for base call quality distribution, percentage bases above Q20 and Q30, GC%, and contamination of sequencing adapter. All the samples have passed the QC threshold of >95%. Further processing of raw sequence reads was performed using fastp^49^ to remove adapter sequences and low-quality bases, followed by excluding the rRNA reads by using bbmap’s bbduk algorithm and SortMeRNA database^50^. The QC passed reads were aligned with indexed Mtb (str. Erdman = ATCC 35801, Assembly:GCA_000668235.1 [Myco_tube_Erdman_V1]) reference genome using HISAT2^51^ aligner, which revealed alignment of ~95.69% of the reads onto the reference genome. Further, the PCR and optical duplicates were removed using Picard tools^52^, and expression values of genes with at least 1 mapped read (*n* = 3964 genes) were obtained as read counts using featureCounts software^53^. Expression similarity between biological replicates was confirmed by spearman correlation and principal components analysis. Biological replicates were grouped as reference (control) and test (*resR/mcdR(−)*) to perform the differential gene expression analysis by edgeR^54^ package after normalizing the data based on trimmed mean of M (TMM) values. Genes that show absolute log2 fold change ≥1, *p* value of ≤0.05, and false discovery rate (FDR) of 5% were considered significant. The expression profile of differentially expressed genes across the samples is presented in volcano plots and heatmap.

### EMSA

DNA probes used for EMSA were prepared by PCR amplifying the desired sequences, using specific primer pairs (Supplementary Data 4) followed by purification of linear DNA from agarose gels using a gel extraction kit (Qiagen). EMSA was set up in the 15 µl reaction mixture containing 17 nM DNA and purified ResR/McdR in binding buffer (10 mM Tris-HCl, pH 8.0, 25 mM NaCl, 50 mM KCl, 10 mM MgCl_2_, 1 mM DTT and 0.002% dextran sulfate) for 15 minutes at 37 °C. Sample containing DNA without ResR/McdR was simultaneously used as control. The DNA-protein complexes were resolved in a native 6 % polyacrylamide gel (acrylamide: bisacrylamide, 28:1, w/w) in 0.5× Tris/Borate/EDTA buffer at room temperature for 2.5 hours at 65 V using the Mini-PROTEAN Tetra cell apparatus (Bio-Rad). After staining the gel with ethidium bromide, signals were visualized under the UV transilluminator. Each binding experiment was performed in duplicate to ascertain the binding of ResR/McdR with DNA.

### Determination of ResR/McdR-promoter binding kinetics

The gel image of EMSA was scanned by ImageJ software (National Institute of Health, USA), and the signal intensities of the bands were used to determine the fraction of total DNA forming complex at different concentrations of ResR/McdR. The Kinetics of DNA-protein interaction was subsequently analyzed by using GraphPad Prism v7.0e software.

### Construction of GFP reporter plasmid

Using the overlapping 2-step PCR approach, a DNA fragment containing the hybrid sequence of the *P*_*rplN*_ and *gfp* was amplified from Mtb genomic DNA and pBEN plasmid^55^, respectively, using primer pairs listed in Supplementary Data 4, such that the resulting DNA fragment contains the *Nhe I* and *EcoR I* restriction sites. Subsequently, the *P*_*rplN*_-*gfp* fragment was digested with *Nhe I* and *EcoR I* and cloned at *Xba I* and *EcoR I* sites in a replicative Zeo^R^ plasmid, pZeo (a derivative of pJV53^56^ harboring zeocin resistance marker). Similarly, a control plasmid was prepared in which 5’-UTR of *pyrG* was cloned upstream to *gfp* in pZeo. The resulting Zeo^R^ GFP reporter plasmid constructs, annotated as pPro_rplN_-gfp and pPro_pyrG_-gfp, were electroporated in the control and *resR/mcdR(−)* strains of Mtb. The activity of *P*_*rplN*_ and *P*_*pyrG*_ promoters was determined in the Kan^R^-Zeo^R^ control:: pPro_rplN_-gfp/pPro_pyrG_-gfp and *resR/mcdR(−)*::pPro_rplN_-gfp/pPro_pyrG_-gfp strains of Mtb by measuring GFP fluorescence after 4 days of incubation with 50 ng/ml ATc. The fluorescence estimation was performed in a plate reader using a black 96-well plate at the excitation and emission wavelengths of 488 nm and 511 nm, respectively.

### Analysis of metabolites

Metabolites were extracted from the empty vector control, *resR/mcdR(−)* and *resR/mcdR(−)::resR/mcdR* strains of Mtb mc^2^ 7902, after 4 days of incubation with 50 ng/ml ATc using six biological replicates, typically as described earlier^25^. Briefly, extraction of metabolites was performed in 1 ml of 80% ice-cold methanol (in water) by multiple freeze-thaw cycles. Metabolites were vacuum dried and suspended in 15% methanol for mass spectrometry (MS) analysis. Cell lysates were separated by injecting 5 µl samples on ultra-performance liquid chromatography using high strength silica T3 column (100 × 2.1 mm i.d packed with 1.7 µm particles) (Waters Corporation) maintained at 40 °C. 0.1% formic acid in water was used as mobile phase A and 0.1% formic acid in acetonitrile as mobile phase B. Elution was performed at a constant flow rate of 0.3 ml/min using the following elution gradient: 0minunte: 99%A in B; 1 minute: 85%A in B; 4 minute: 65%A in B; 7–9 minute: 5%A in B; and 10–14 minute: 99%A in B. MS was performed using the Orbitrap Fusion mass spectrometer (Thermo Scientific) attached with heated electrospray ionization source on positive (spray voltage of 4000volt) and negative (spray voltage of 35000volt) modes. A 120,000 resolution in MS and 30,000 resolution in data-dependent MS2 scan modes were used keeping the sheath gas setting at 42 and auxiliary gas setting at 11. The mass scan range of 50–1000 m/z was set with automatic gain control (ACG) target of 200,000 ions and maximum injection time of 80 milliseconds for MS, and ACG target of 20,000 ions keeping the same maximum injection time for MS/MS.

After acquisition, data were processed with the help of compound discoverer 2.1 software (Thermo Scientific) using default settings for metabolite identification and quantitation. Data acquired with blank runs were used for removing the background noise arising from the mobile phase. Identification of metabolites was performed primarily on the basis of in-house metabolite standards using accurate mass, fragmentation pattern, and retention time information by spectral matching on the mzCloud database, available with compound discoverer 2.1.

### Surface sensing of translation (SUnSET)

After 7 days of treatment with 50 ng/ml ATc, cultures of control, *resR/mcdR(−)* and *resR/mcdR(−)::resR/mcdR* strains were pelleted and suspended in the fresh medium as 1.0 OD_600_/ml. Cultures were incubated with 50 µg/ml puromycin for 1 hour at 37 °C, followed by centrifugation to pellet the cultures. Whole cell lysates were prepared by bead-beating, as described above, and 30 µg protein samples were resolved by denaturing SDS-PAGE using 10% gel and transferred to nitrocellulose membrane by semi-dry electro-transfer method. Immunoblotting was performed using 1:10,000 dilution of anti-puromycin antibodies, as suggested by the manufacturer (Merck). Blot was incubated with Super Signal West Femto (Thermo Fisher) and chemiluminescence signals were obtained with the help of a gel documentation system (Bio-Rad).

### Ribosome profiling

Ribosomes were purified from the empty vector control, *resR/mcdR(−)* and *resR/mcdR(−)::resR/mcdR* after 7 days of incubation with 50 ng/ml ATc, and profiling of ribosome was performed by ultracentrifugation of equal amount of crude ribosome preparations on the linear gradient of sucrose, typically as described earlier^26^. Briefly, the cell pellet was lysed in the lysis buffer-A (20 mM HEPES pH 7.4, 20 mM MgCl_2_, 100 mM NH_4_Cl, and 3 mM DTT) containing 1× protease inhibitor cocktail (Merck), by using Mixer Mill 500 (Retsch). The cell lysate was centrifugation at 13,000 rpm for 30 minutes at 4˚C, and the supernatant was layered on 1.1 M sucrose cushion and centrifuged at 100,000 × *g* at 4˚C for 13 hours using P70AT2 rotor (Hitachi). The pellet which contains the crude ribosomes was suspended in the lysis buffer-A. The concentration of crude ribosome was estimated by measuring absorbance at 260 nm (A_260_). Equal A_260_ units of samples from control, *resR/mcdR(−)* and *resR/mcdR(−)::resR/mcdR* strains were layered on 10–50% linear gradient of sucrose and centrifuged at 38,000 rpm for 4.5 hour at 4˚C in P40ST rotor (Hitachi). The gradients were fractionated using a piston gradient fractionator (Biocomp Instruments, Canada) and A_260_ was estimated for each fraction.

### Scanning electron microscopy (SEM)

To prepare bacterial cultures for SEM analysis, both the control and *resR/mcdR(−)* strains were treated with 50 ng/ml of ATc for 4 days. Cells were pelleted and washed twice with 0.10 M phosphate buffer (pH 7.4). The resulting pellets were resuspended in Karnovsky fixative and passed through 24 G needle 10 times to break the clumps. After overnight incubation at 4 °C, the fixed cells were washed twice with 0.1 M phosphate buffer and dehydrated by washing through gradient of acetone (30%, 50%, 70%, 80%, 90%, and 100%) at 2000rpm, 25 °C, 10 minutes each. dehydrated cells were then coated with HMDS overnight in a desiccator, on a cover slip. The next day samples were sputter coated with gold and observed under FEI Apreo Volumescope microscope in the Advanced Technology Platform Centre at the Regional Centre for Biotechnology, Faridabad, India.

### Time-kill kinetics assay

Cultures of empty vector control and the respective knockdown strains were grown in a broth medium in the presence or the absence of ATc for four days. Subsequently, all the cultures were diluted in the respective media to OD_600_ of 0.05 followed by incubation with antibiotics at a final concentration of 10× MIC. Aliquots of the bacterial cultures, left untreated, were used as drug-free controls. Bacterial viability was determined by CFU plating on days 0, 1, 3, and 5, post-antibiotic treatment after washing a portion of bacterial pellet twice with 1× PBS to remove residual antibiotics. Enumeration of bacterial CFU was performed by counting CFUs in dilutions yielding 10–100 visually separated colonies. Percentage killing was determined at each time point with respect to day 0 CFU.

### Estimation of PAE

For estimating the PAE, a series of fivefold dilutions of the bacterial cultures, after 24 hours of treatment with drugs at a final concentration of 10× MIC, were prepared in PBS. From each dilution 4 µl was spotted on a 7H11-OADS agar plates without antibiotics, and spotting was observed till 21 days after the first appearance of bacterial colony. Dilutions yielding similar profiles of ATc-treated and untreated cultures in the absence of drug, were considered for determining the PAE after drug exposure.

### Statistics and reproducibility

Some experiments were only performed with three technical replicates, or two biological replicates. Statistical analysis was performed with data obtained from three or more biological repeats by determining *p* values using the two-stage setup method of Benjamini, Krieger, and Yekutieli keeping FDR (*Q*) of 1% with the help of GraphPad Prism v7.0e software.

### Reporting summary

Further information on research design is available in the Nature Portfolio Reporting Summary linked to this article.

## Supplementary information

Supplementary Information

Description of Additional Supplementary Data

Supplementary Data 1

Supplementary Data 2

Supplementary Data 3

Supplementary Data 4

Reporting Summary

## Data Availability

The raw RNASeq data are available from the GEO database under project accession number GSE214729. Source data underlying figures are provided in Supplementary Data 1.
