# Supplementary material for: ResR/McdR-regulated protein translation machinery contributes to drug resilience in Mycobacterium tuberculosis
Source: Commun Biol. 2023 Jul 11;6:708. doi: 10.1038/s42003-023-05059-8 (PMC10336103; doi:10.1038/s42003-023-05059-8)

Supplementary Information

ResR/McdR-regulated protein translation machinery contributes to drug resilience in *Mycobacterium tuberculosis*

Pramila Pal[1,2,4], Mohd Younus Khan[1,2,4], Shivani Sharma[3], Yashwant Kumar[1], Nikita Mangala[1,2], Prem S. Kaushal[3] and Nisheeth Agarwal[1]*

[1]Translational Health Science and Technology Institute, NCR Biotech Science Cluster, 3rd Milestone, Faridabad–Gurgaon Expressway, Faridabad- 121001 (Haryana), India

[2]Jawaharlal Nehru University, New Mehrauli Road, New Delhi- 110067 (Delhi), India

[3]Regional Centre for Biotechnology, NCR Biotech Science Cluster, 3rd Milestone, Faridabad–Gurgaon Expressway, Faridabad- 121001 (Haryana), India

[4]These authors contributed equally: Pramila Pal, Mohd Younus Khan.

*Correspondence author: Nisheeth Agarwal

E-mail: nisheeth@thsti.res.in

Supplementary Figure 1 Alignment of Mtb ResR/McdR with its counterparts from actinobacteria. Shown is the multiple sequence alignment of ResR/McdR from Mtb Erdman (Query_19538) with its counterparts from various actinobacteria. Alignment was performed using blastp tool of NCBI (https://blast.ncbi.nlm.nih.gov/Blast.cgi?PAGE=Proteins) keeping default parameters. Numbers above the alignment indicate positions of the amino acid residues in the query sequence. Position of the HTH domain in the Mtb ResR/McdR is marked. “+” in the parentheses indicates orientation of the respective ORFs. Red bars indicate highly conserved residues, blue bars represent lower conservation and gray bars indicate mismatches.

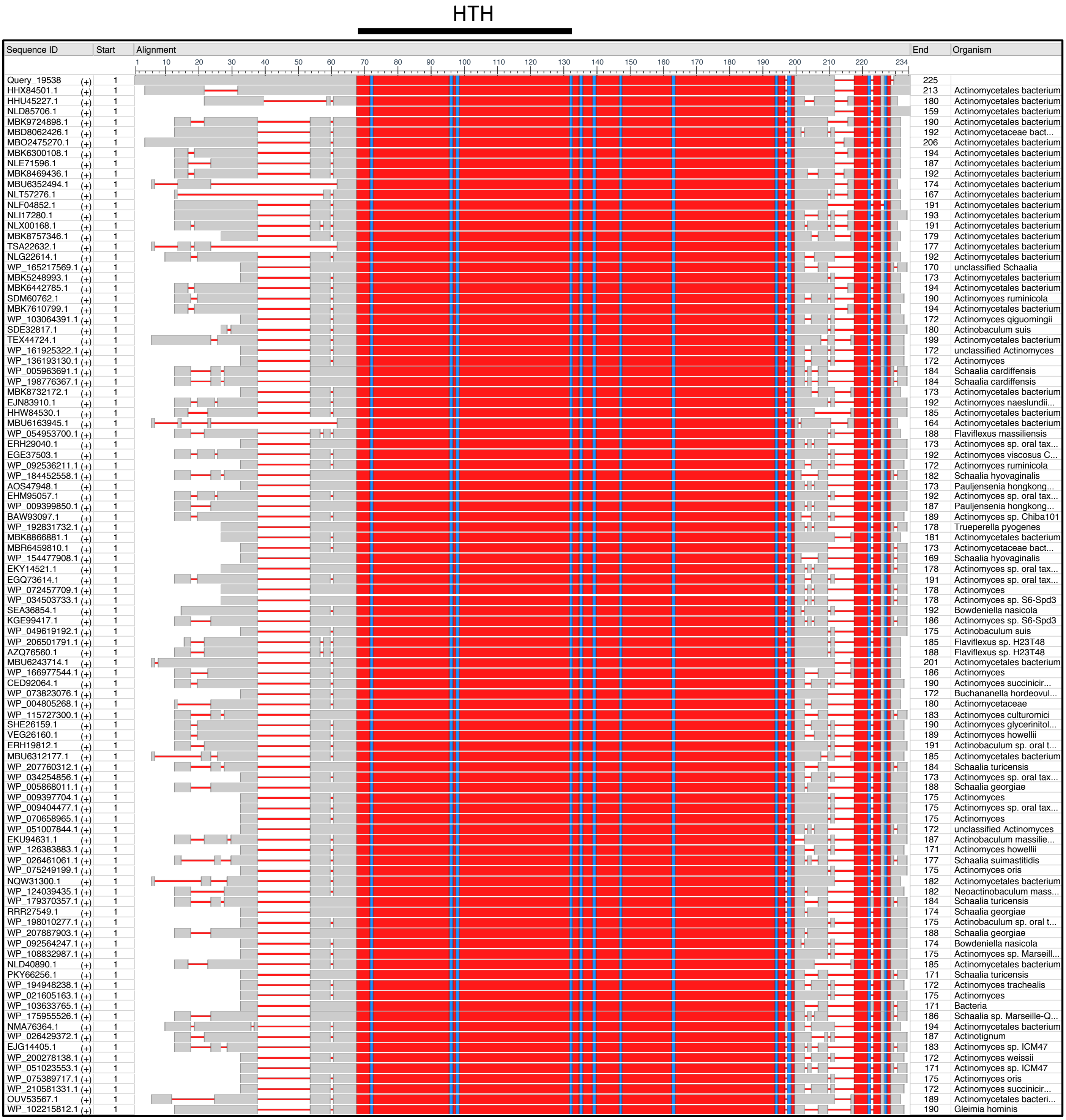

Supplementary Figure 2 Alignment of Mtb ResR/McdR with homologous proteins in Mtb-complex bacteria. Shown is the multiple sequence alignment of ResR/McdR from Mtb Erdman (Query_81548) with its homologues from Mtb-complex bacteria. Alignment was performed using blastp tool of NCBI (https://blast.ncbi.nlm.nih.gov/Blast.cgi?PAGE=Proteins) keeping default parameters. Numbers above the alignment indicate positions of the amino acid residues in the query sequence. "+" in the parentheses indicates orientation of the respective ORFs Red bars indicate highly conserved residues and gray bars indicate mismatches.

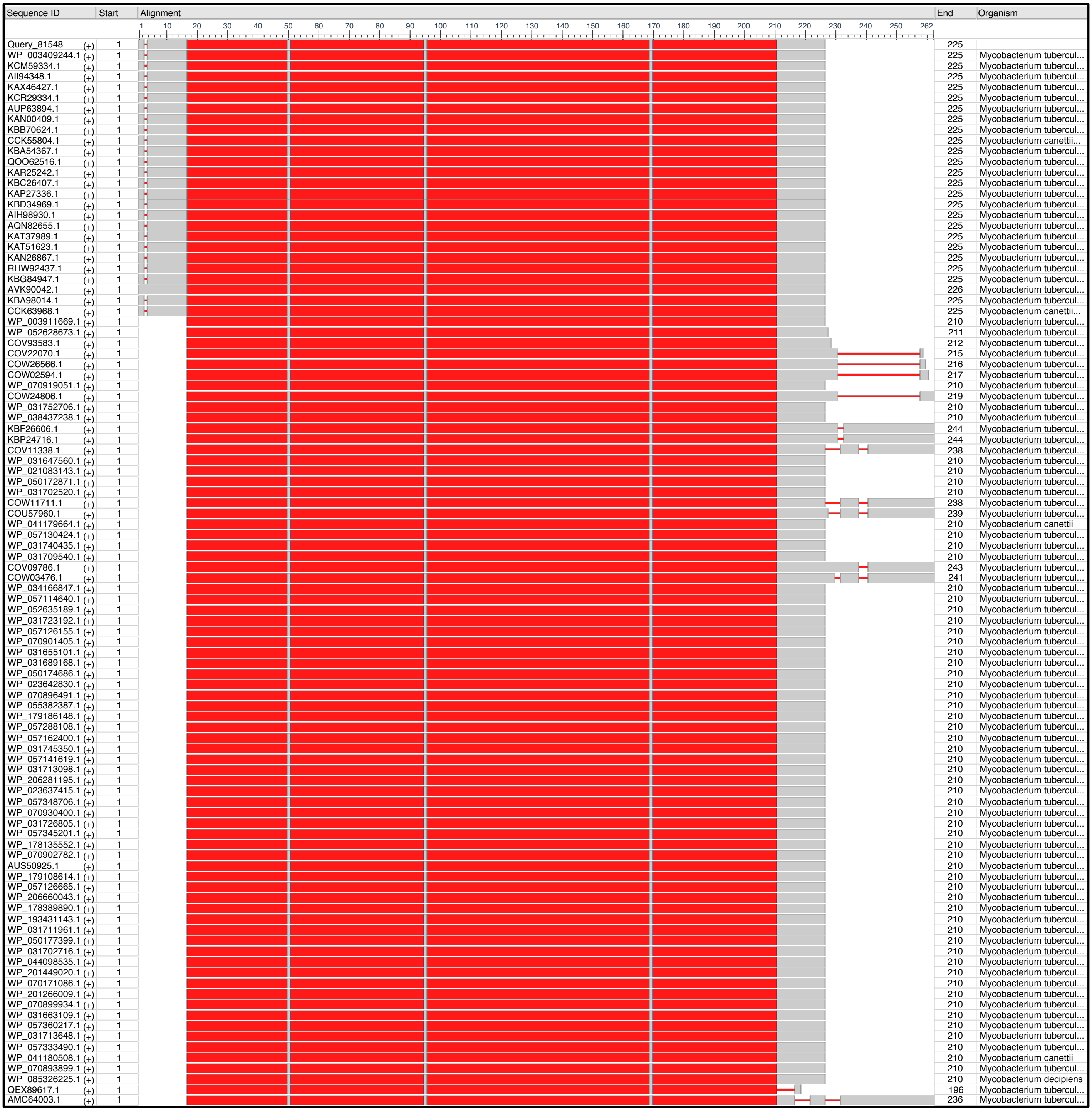

Supplementary Figure 3 Analysis of ResR/McdR conformation. a *In silico* analysis of ResR/McdR. Analysis of ResR/McdR by d2p2 (https://d2p2.pro/) reveals the presence of a putative DNA-binding HTH domain in the middle (red) from 65-137aa (red) and disordered regions (green) comprising of 3-51aa at the N-terminus and 201-225aa at the C-terminus. **b** Analysis of purified 6xHis-ResR/McdR by size exclusion chromatography. The graph shows the absorbance of protein at 280nm (mAU) in different elution fractions (top). Molecular mass was determined by using known standards, as described in the Methods. Samples were also resolved on denaturing polyacrylamide gels to detect protein in the corresponding elution fractions upon Coomassie Brilliant Blue staining (bottom). Broken lines show the fractionation range of the column.

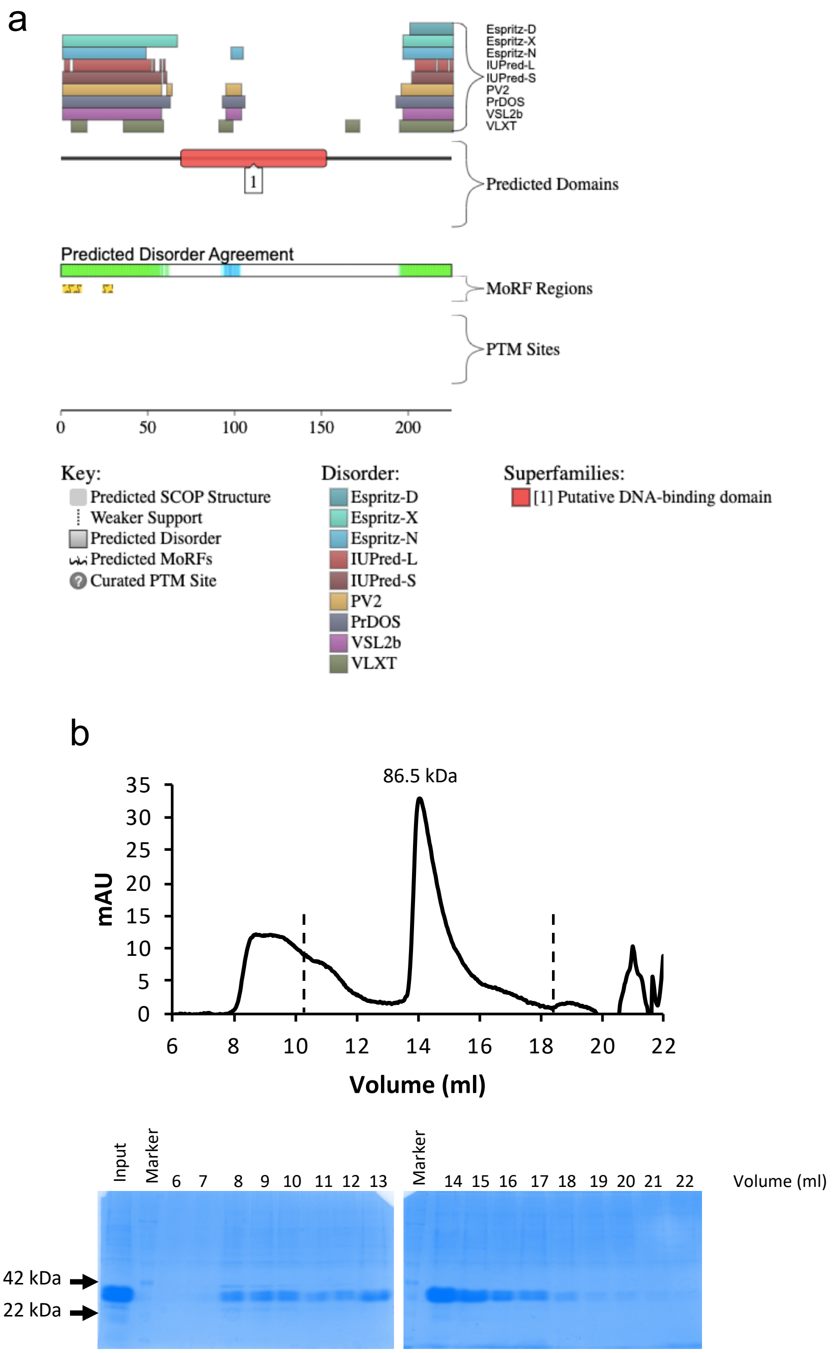

Supplementary Figure 4 Expression analysis of *resR/mcdR* in Mtb. a Locus analysis of *resR/mcdR*. *resR/mcdR* is situated in close proximity to the two other genes, *ERDMAN_2021* and *gcvB*. Coloured bars above the ORFs represent PCR amplicons of the junction sequences by respective primer pairs. **b** Examination of *resR/mcdR* transcript expression. PCR amplification of the junction sequences with the complementary DNA (cDNA) template using different primer pairs, as shown in **a**, reveals amplification of only internal sequence with F-R1 primer pair, whereas other primer sets (F-R2 and F-R3) were unable to amplify the respective sequences from cDNA, thus suggesting that *resR/mcdR* expression is independent of the downstream genes in its locus. Reactions with genomic DNA (gDNA) template as well as without RT enzyme (-RT) were used as positive and negative controls, respectively. **c** *In vitro* growth analysis of Mtb. The graph shows different growth stages of the wild-type Mtb Erdman, as estimated by OD_{600} measurement of the bacterial culture. **d** Expression analysis of ResR/McdR by immunoblotting. Expression of ResR/McdR was analysed in Mtb Erdman by anti-ResR/McdR immunoblotting of the whole cell lysates prepared from bacteria at different stages of growth, shown in **c**. After protein transfer, membrane was stained with ponceau S dye to ascertain equal loading of samples. The ResR/McdR-specific signal is marked by the arrow on the right. n.s. represents non-specific signals by anti-ResR/McdR antibodies, present in all the samples in **d**. The arrows on the left indicate the positions of molecular weight markers. Molecular weight markers were accentuated by hand as the signal faded after several washes of the blot. kDa, kilo Dalton.

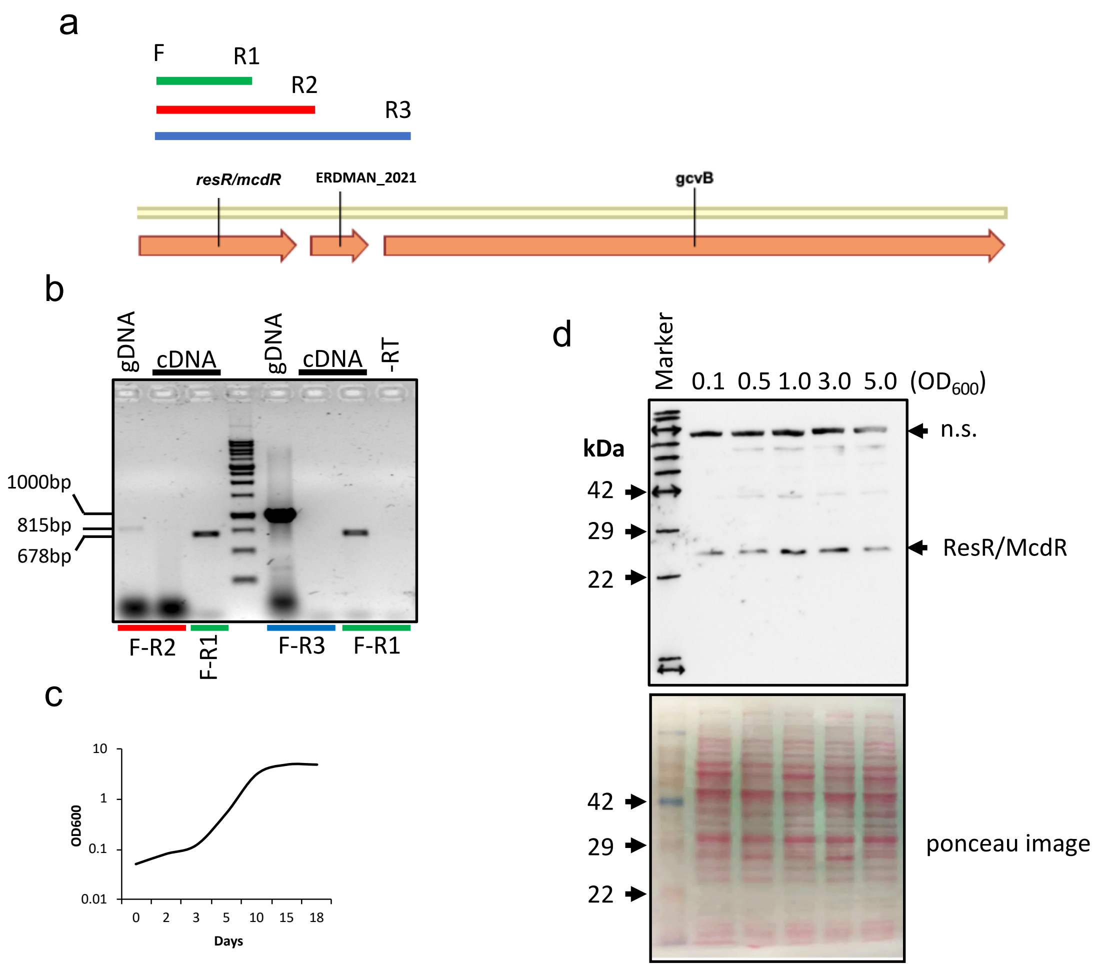

Supplementary Figure 5 Determination of TSS of *resR/mcdR* by 5' RACE. Mapping of transcriptional start site (TSS) of *resR/mcdR* by 5' rapid amplification of cDNA end (5' RACE). The chromatogram shows the results of Sanger sequencing. The corresponding DNA sequence of the sense strand is displayed above the chromatogram. TSS is identified as the first base in the 5'-UTR of *resR/mcdR* following the long stretch of 'G' residues. Position of TSS is marked by the arrow.

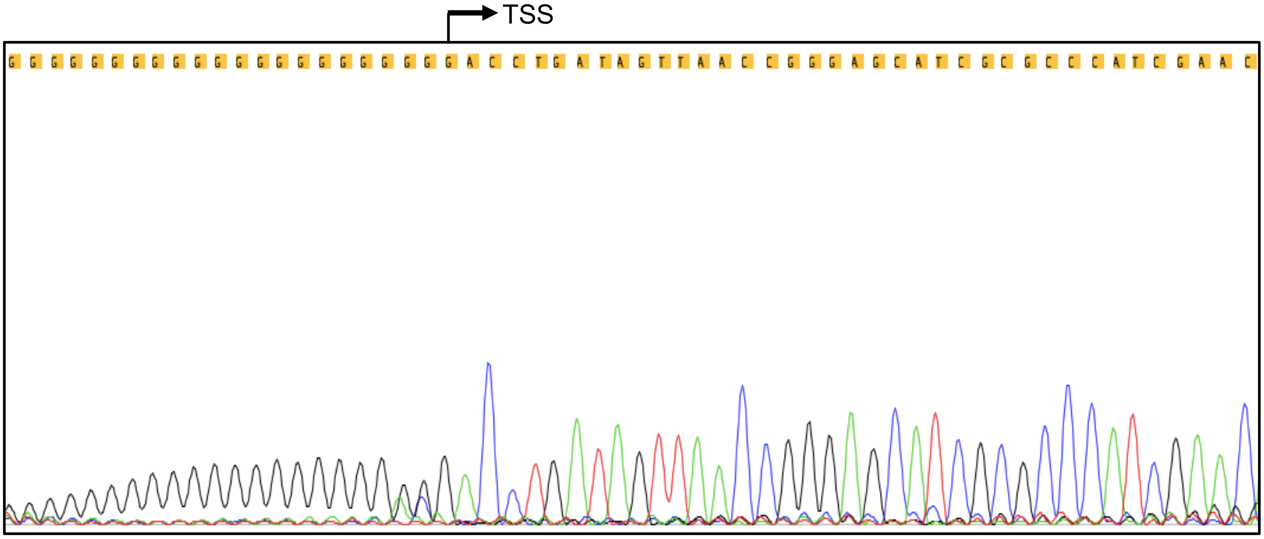

Supplementary Figure 6 Optimization of ATc dose for suppression of *resR/mcdR.* **a** Validation of CRISPRi-mediated silencing of *resR/mcdR* by qRT-PCR. The graph shows relative expression of *resR/mcdR* transcripts in *the* knockdown strain of Mtb Erdman at different ATc concentrations compared to their levels in the ATc-untreated control. **b** Analysis of *dcas9* expression. Shown are the relative expression levels of d*cas9* transcripts in the *resR/mcdR(-)* strain at different ATc concentrations compared to their levels in the ATc untreated control. Values were obtained after normalization with the expression of a constitutive gene in the respective samples. **c**, **d** Effect of ATc dose-dependent suppression of *resR/mcdR* on *in vitro* growth of Mtb. Growth of *resR/mcdR(-)* was compared after 4 days in the absence or the presence of different concentrations of ATc by measuring OD_{600} (**c**) and by CFU estimation (**d**). ATc treatment was performed in all the experiments at the initial culture OD_{600} of 0.05. Data represent mean $\pm$ s.d. (shown by error bars) from multiple (n=3) biological repeats in **a**, **b**, **d**. Mean values from multiple (n=3) measurements are shown in **c**. *p* values in **a**, **b**, **d** were obtained for the respective samples after comparison with ATc-untreated sample, as described in Methods.

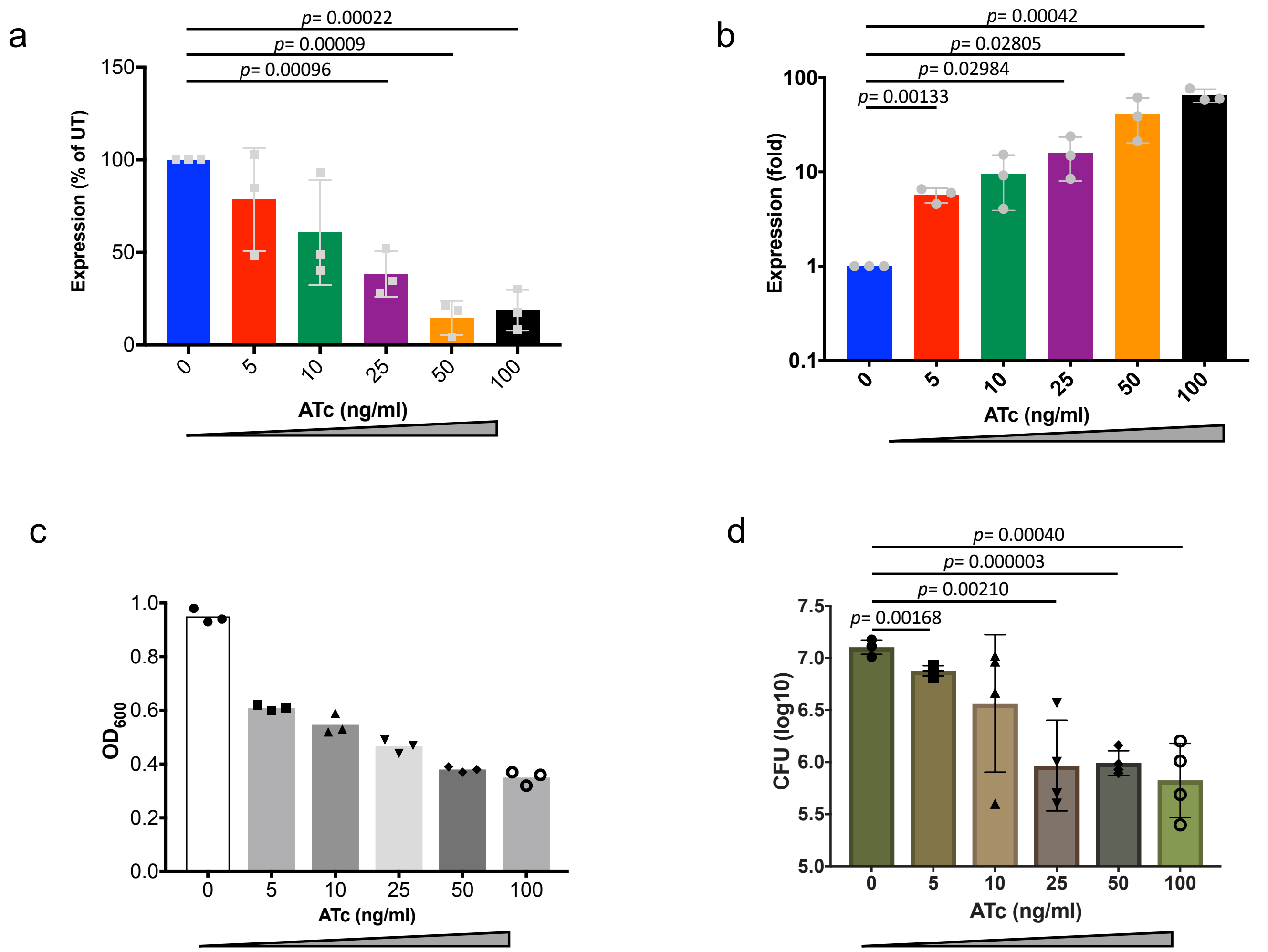

Supplementary Figure 7 Analysis of ResR/McdR expression by immunoblotting. Uncropped image of the blot shown in Figure 1. a Anti-ResR/McdR immunoblot of whole cell lysates from the *resR/mcdR(-)*, control, and *resR/mcdR(-)::resR/mcdR* strains of Mtb. **b** Loading profile of samples by ponceau S staining. Equal loading of samples is confirmed by ponceau S staining of the membrane before probing with the anti-ResR/McdR antibodies. n.s. represents non-specific signals by Anti-ResR/McdR antibodies, present in all the samples in **a**. Boxed areas in **a**, **b** show blot regions in Figure 1. kDa, kilo Dalton

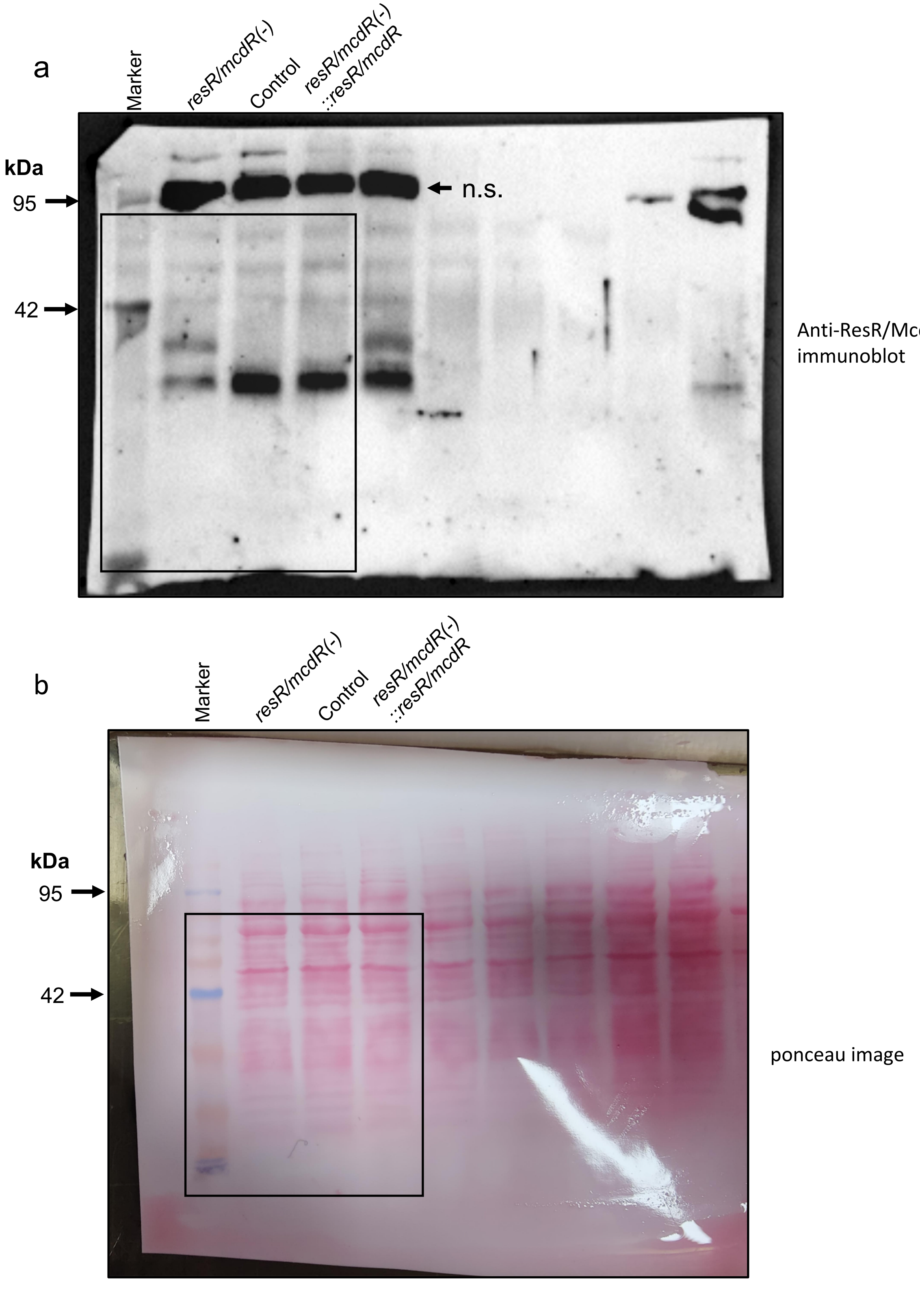

Supplementary Figure 8 Effect of *resR/mcdR* silencing on bacterial cell length. a Analysis of bacterial morphology by electron microscopy. Both control and *resR/mcdR(-)* strains of Mtb Erdman were analysed by scanning electron microscopy (SEM) to examine the cell length of these bacteria. Shown is the SEM image from a representative field for each strain at 25000x magnification. Scale bar is shown for size reference. **b** Effect of *resR/mcdR* silencing on bacterial cell length. Scatter dot plot exhibits the mean $\pm$ s.d. (shown by error bars) length of the control and the knockdown strains, as estimated from multiple (n=150) randomly selected bacteria. *p* value in **b** was obtained for the knockdown strain after comparison with control, by Welch's t-test using GraphPad Prism v7.0e software.

a

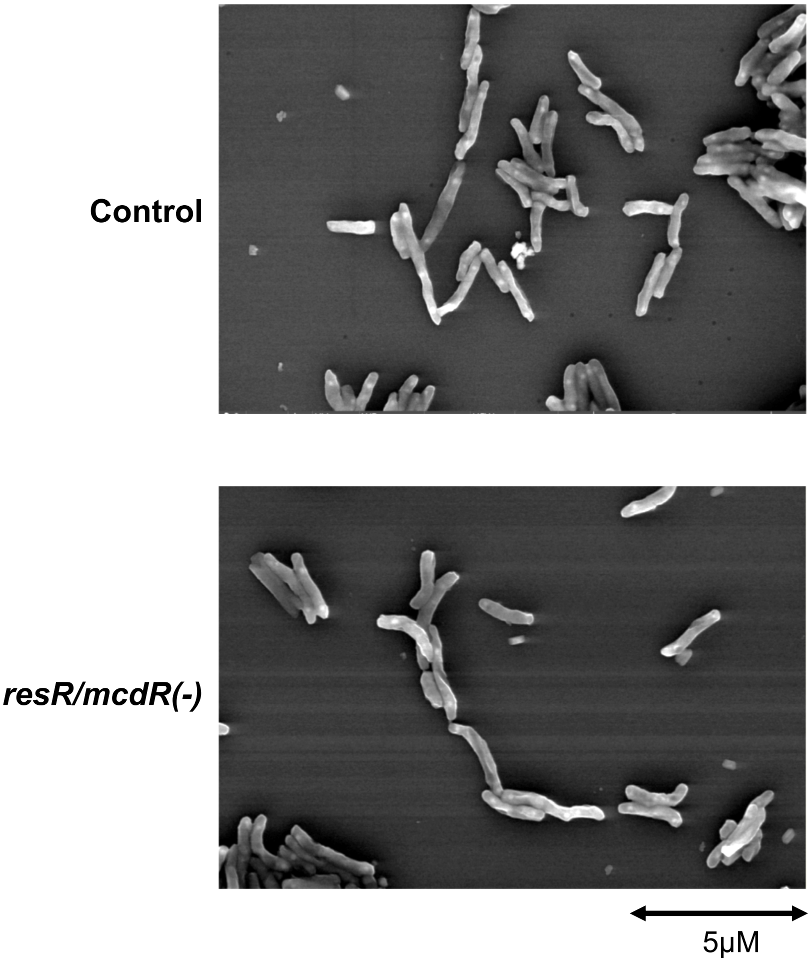

b

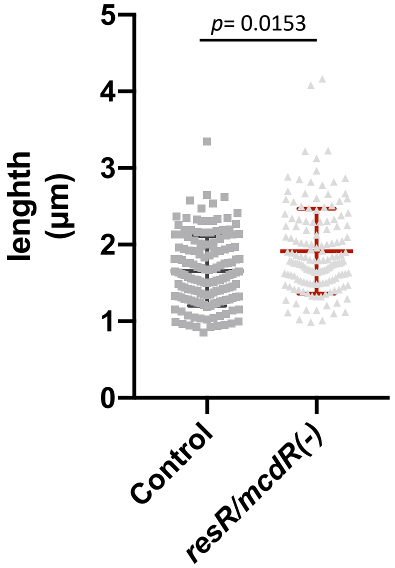

Supplementary Figure 9 Intracellular survival of control strain of Mtb Erdman in mouse organs. a, **b** Comparative analysis of growth of the empty vector control strain in mouse lungs (**a**) and spleen (**b**) upon treatment with doxycycline+5% sucrose (+Doxy) or 5% sucrose (-Doxy), respectively. Bacterial loads in lungs and spleen were estimated at the designated time points by CFU enumeration, as described in the Methods. Data represent mean ± s.d. (shown by error bars) from multiple (n=4) animals in **a**, **b**.

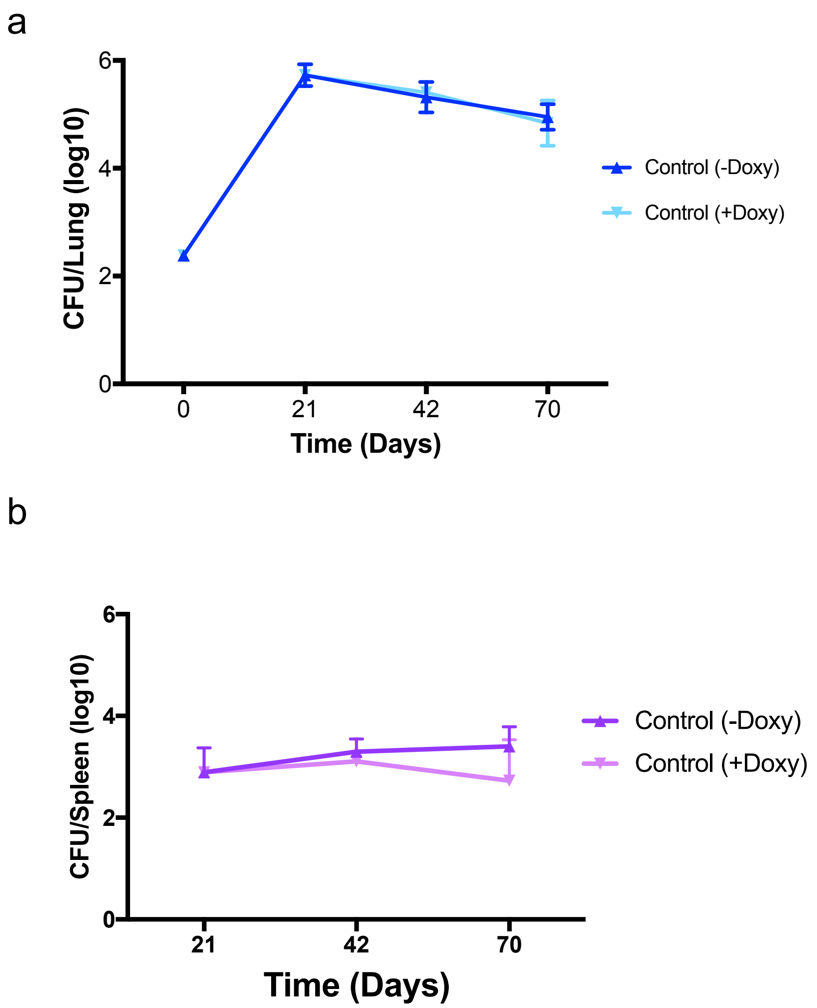

Supplementary Figure 10 Effect of *resR/mcdR* silencing on initiation of protein synthesis in Mtb. Uncropped image of the blot shown in Figure 5. a Anti-puromycin immunoblot of whole cell lysates. Empty vector control, *resR/mcdR(-)* and *resR/mcdR(-)::resR/mcdR* strains of Mtb Erdman were incubated with 50µg/ml puromycin for 1 hour after 7 days of ATc treatment. Lysates prepared from the respective strains were subjected to anti-puromycin immunoblotting, which reveals significant inhibition of newly translated proteins upon *resR/mcdR* silencing, which is restored by complementation with the wild-type copy of *resR/mcdR*. **b** Loading profile of samples by ponceau S staining. Equal loading of samples is confirmed by ponceau S staining of the membrane before probing with the anti-puromycin antibodies. The arrows on the left in **a**, **b** indicate the positions of molecular weight markers. Molecular weight markers were accentuated by hand as the signal faded after several washes of the blot. Boxed areas in **a**, **b** show blot regions in Figure 5. kDa, kilo Dalton.

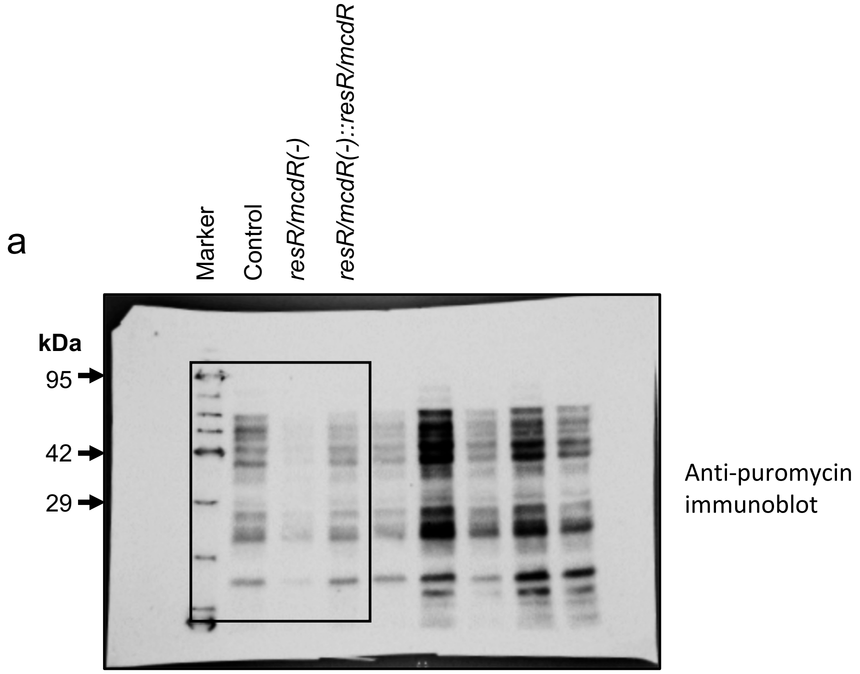

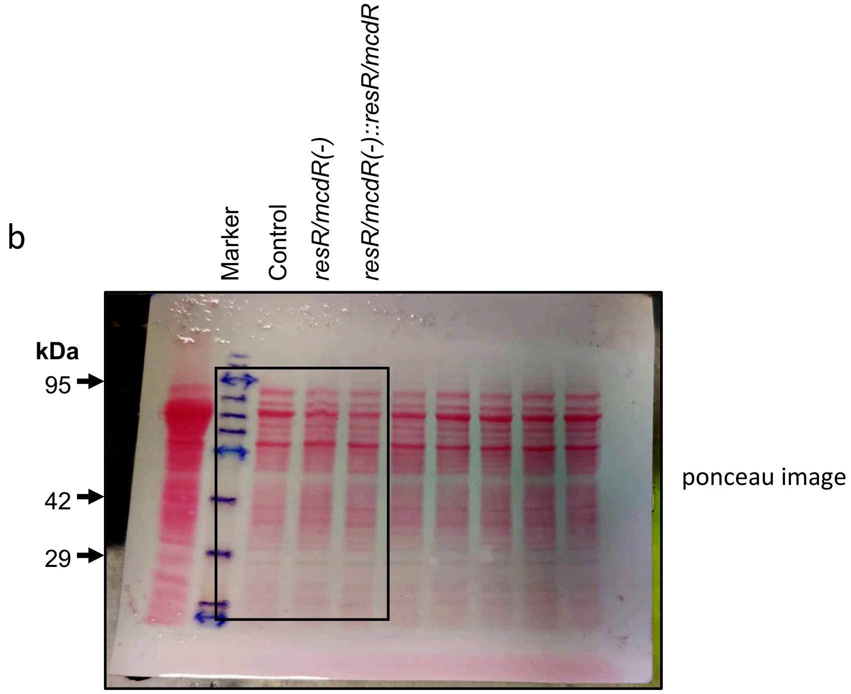

Supplementary Figure 11 Analysis of full-length and truncated derivative of Mtb ResR/McdR. a SDS-PAGE analysis of purified proteins. Shown are the Coomassie Brilliant Blue-stained denaturing polyacrylamide gels with different elution fractions of the wild-type and the N-terminal truncated derivatives of GST-tagged Mtb ResR/McdR. Prestained molecular mass markers were simultaneously used for determination of protein sizes in the eluted fractions. **b** Analysis of the secondary structure of purified proteins by circular dichroism (CD) spectroscopy. **c** Percentage of different secondary structure components. Different secondary structure components in the respective proteins were determined by using the BeStSel server (http://bestsel.elte.hu). kDa, kilo Dalton.

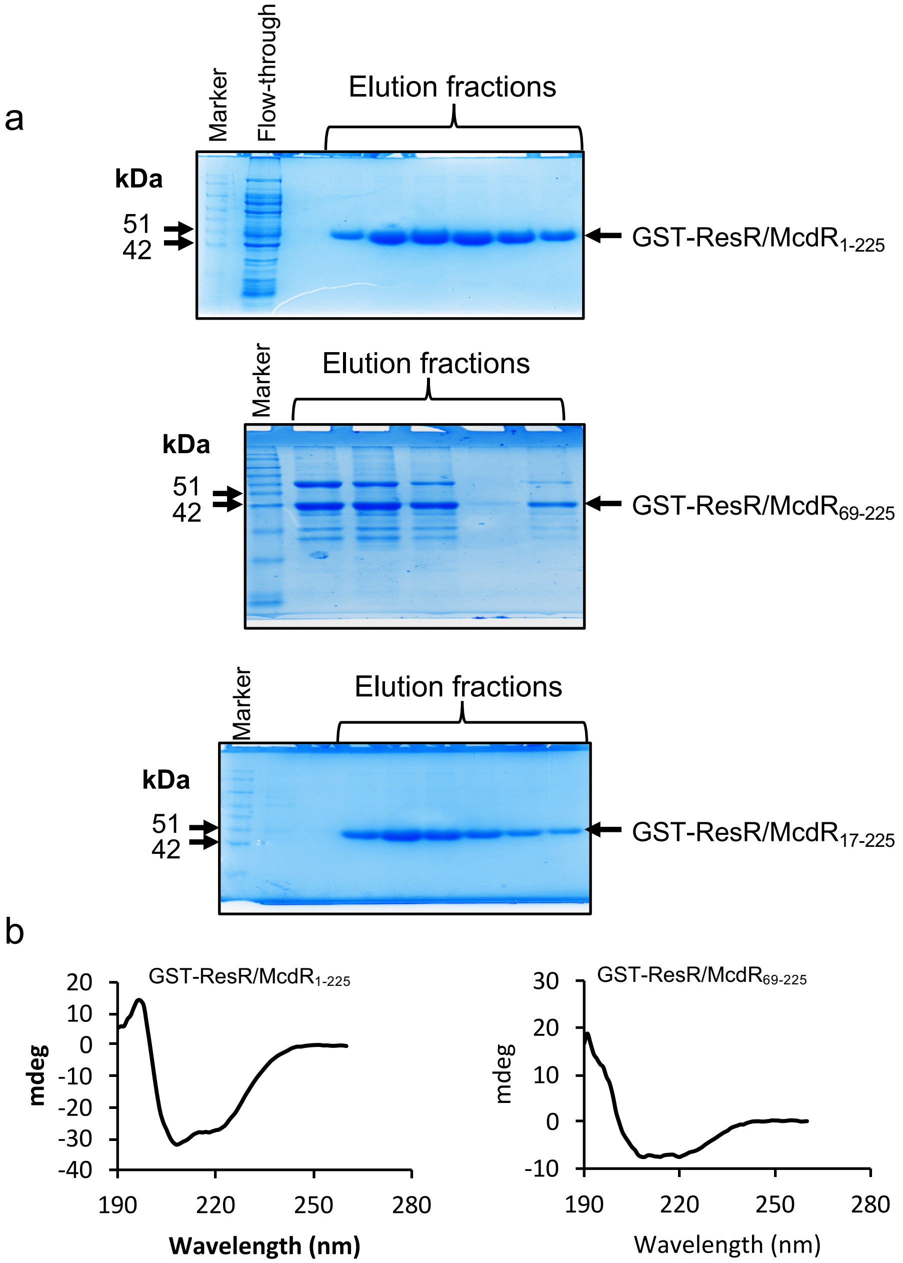

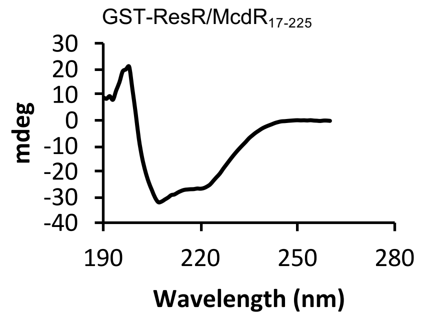

c

	[1-225]	[69-225] (%)	[17-225]
Helix:	17.2	0.1	16.8
β sheets:	28.7	45.1	28.6
Turn:	14.0	14.3	13.9
Others:	40.1	40.5	40.7

Supplementary Figure 12 Analysis of ResR/McdR binding with *whiB2* promoter. a Analysis of P_{whiB2} sequence used in EMSA. The TSS site is marked by bent arrow and the underlined sequences represent -35 and -10 promoter elements. Position of the full-length (P_{whiB2_FL}) and the truncated version (P_{whiB2_TR}) of P_{whiB2} is shown by double headed arrows. The putative ResR/McdR-recognition sequence in the P_{whiB2_FL} is shown in black box. The conserved residues are highlighted in bold-face type. **b** Analysis of ResR/McdR binding with P_{whiB2} by EMSA. Binding was performed by using different concentrations of ResR/McdR with P_{whiB2_FL}, which reveals ResR/McdR dose-dependent complex formation with the full-length promoter. Notably, absence of complex with P_{whiB2_TR}, even at a maximum concentration of the protein confirms the sequence-specific binding of ResR/McdR with the recognition sequence in P_{whiB2_FL} promoter. **c** Analysis of ResR/McdR-P_{whiB2_FL} binding kinetics. The graph shows percentage of total DNA probe forming complex at the respective concentrations of ResR/McdR, as shown in the panel **b**. The dissociation constant (K_d) was determined by using GraphPad Prism v7.0e software. Non-linear fit of data from multiple (n= 2) biological repeats is shown in **c**.

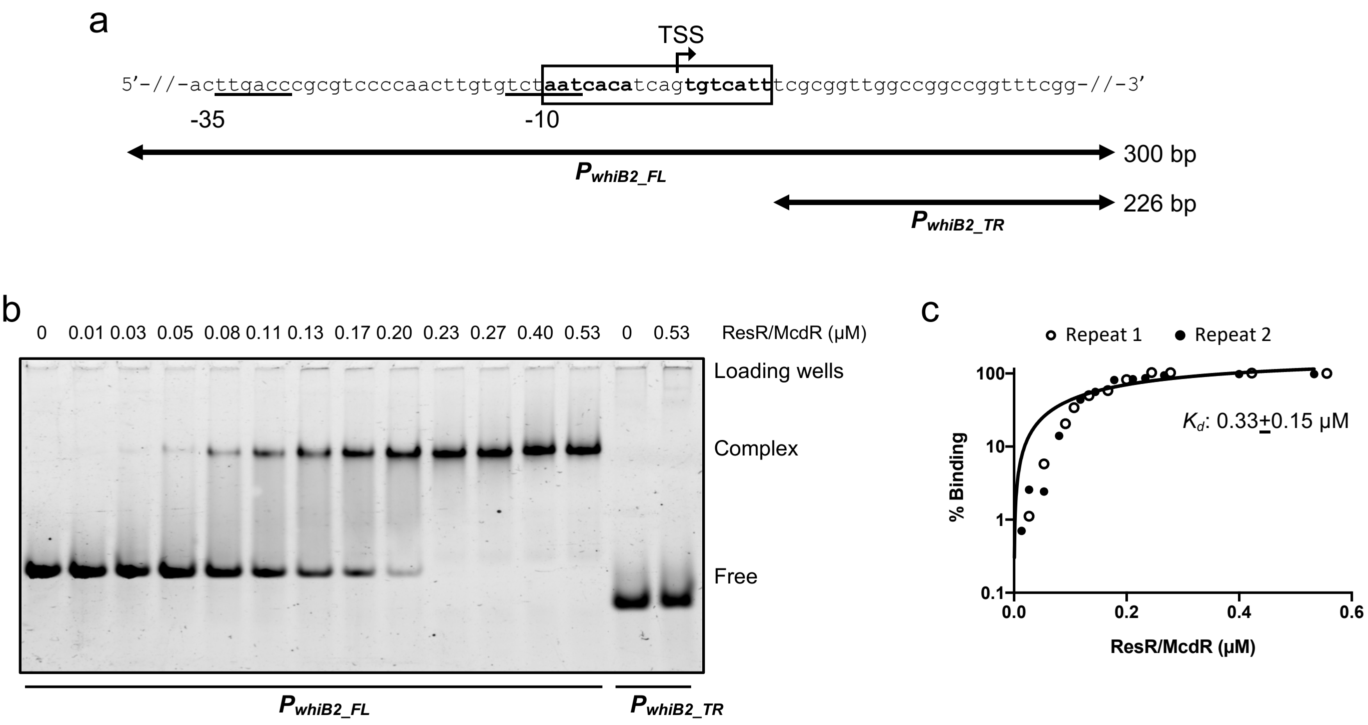

Supplementary Figure 13 Analysis of *rplN* locus in Mtb. Shown is the arrangement of genes in the *rplN* locus of Mtb Erdman, which are transcribed as operon from a common promoter upstream to *rplN*. Positions of -35 and -10 elements in the promoter region and the TSS are shown.

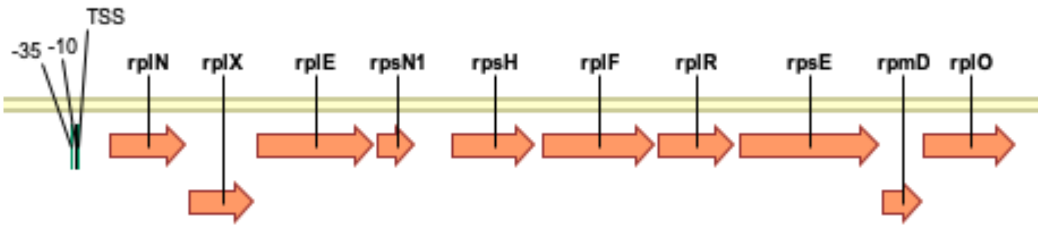

Supplementary Figure 14 Analysis of DNA-binding activity of ResR/McdR homologue from Msm. a Analysis of the purified GST-MSMEG_3644 (ResR/McdR^{MS}) by SDS-PAGE. **b** Analysis of the secondary structure of purified protein by circular dichroism spectroscopy. Different secondary structure components in ResR/McdR^{MS} were determined by using the BeStSel server (http://bestsel.elte.hu). **c** Analysis of ResR/McdR^{MS} binding with P_{rplN} by EMSA. Binding was performed by using different concentrations of ResR/McdR^{MS} with P_{rplN_FL}, which reveals ResR/McdR^{MS} dose-dependent complex formation with the full-length promoter fragment. Notably, absence of complex with P_{rplN_TR}, even at a maximum concentration of the protein confirms the sequence-specific binding of ResR/McdR^{MS} with the recognition sequence in P_{rplN_FL} promoter. **d** Analysis of ResR/McdR^{MS}-P_{rplN_FL} binding kinetics. The graph shows percentage of total DNA probe forming complex at the respective concentrations of ResR/McdR^{MS}, as shown in **c**. The dissociation constant (K_d) was determined by using GraphPad Prism v7.0e software. Non-linear fit of data from multiple (n= 2) biological repeats is shown in **d**. kDa, kilo Dalton.

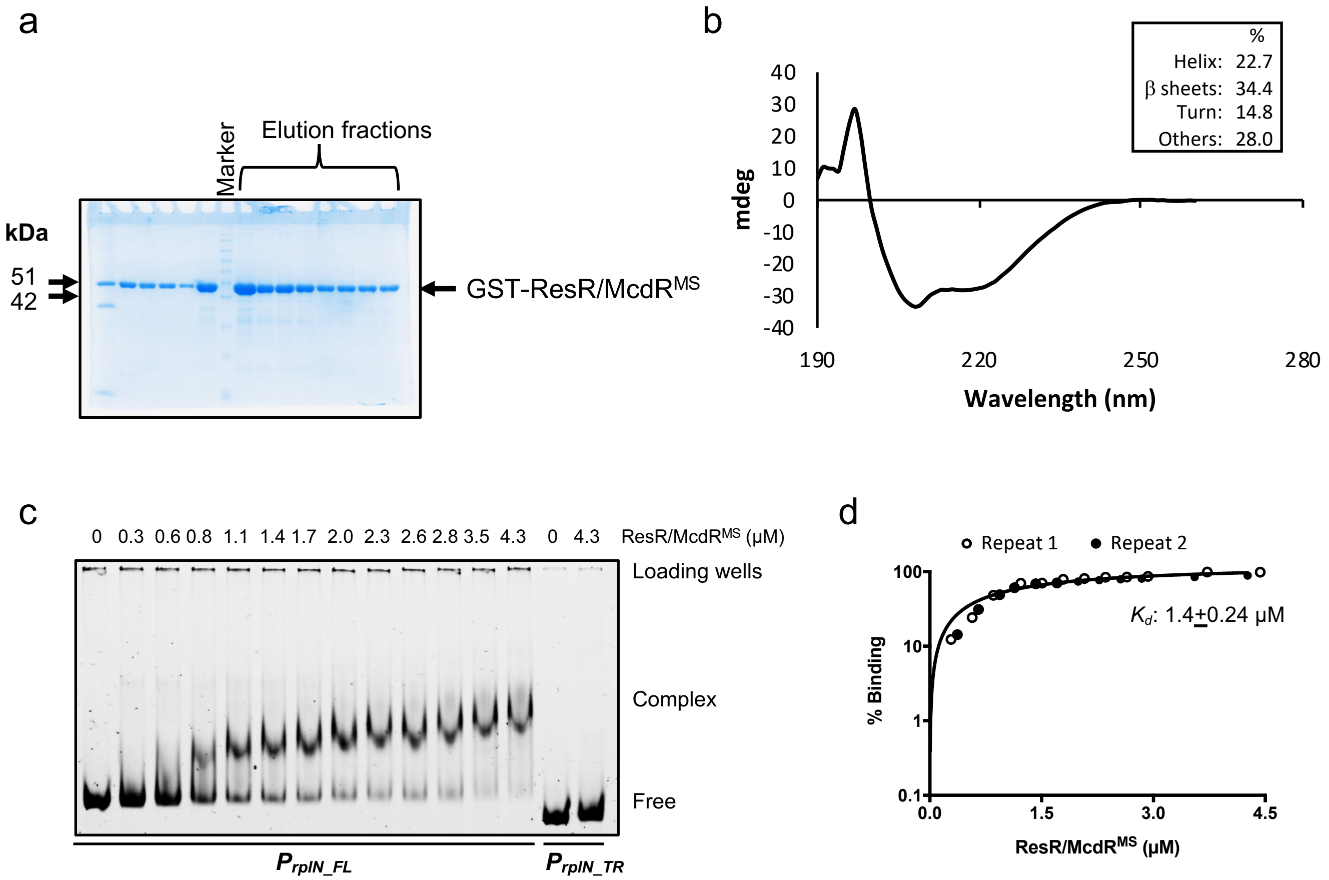

Supplementary Figure 15 PAE with the control and *whiB2(-)* strains of Mtb. a Time-kill kinetics of the empty-vector control. Time-kill kinetics was examined in response to treatment with isoniazid (INH), rifampicin (RIF), streptomycin (STR), and levofloxacin (LEV). The MDK_{99} by each drug, after exposure to 10X MIC is depicted by the dashed line. **b** Post-antibiotic recovery dynamics of the control Mtb. Representative images, captured between 13-21 days of incubation, depict the post-antibiotic recovery dynamics of the control strain of Mtb. **c** Time-kill kinetics for the *whiB2(-)*. Time-kill kinetics of *whiB2(-)* was examined in response to treatment with 10X MIC of different drugs, as mentioned in **a**. The MDK_{99} by each drug is depicted by the dashed line. **d** Post-antibiotic recovery dynamics of *whiB2(-)*. Representative images, captured between 13-21 days of incubation, depict the post-antibiotic recovery dynamics of Mtb depleted with *whiB2*. Scale bars in **b**, **d** are shown for size reference.

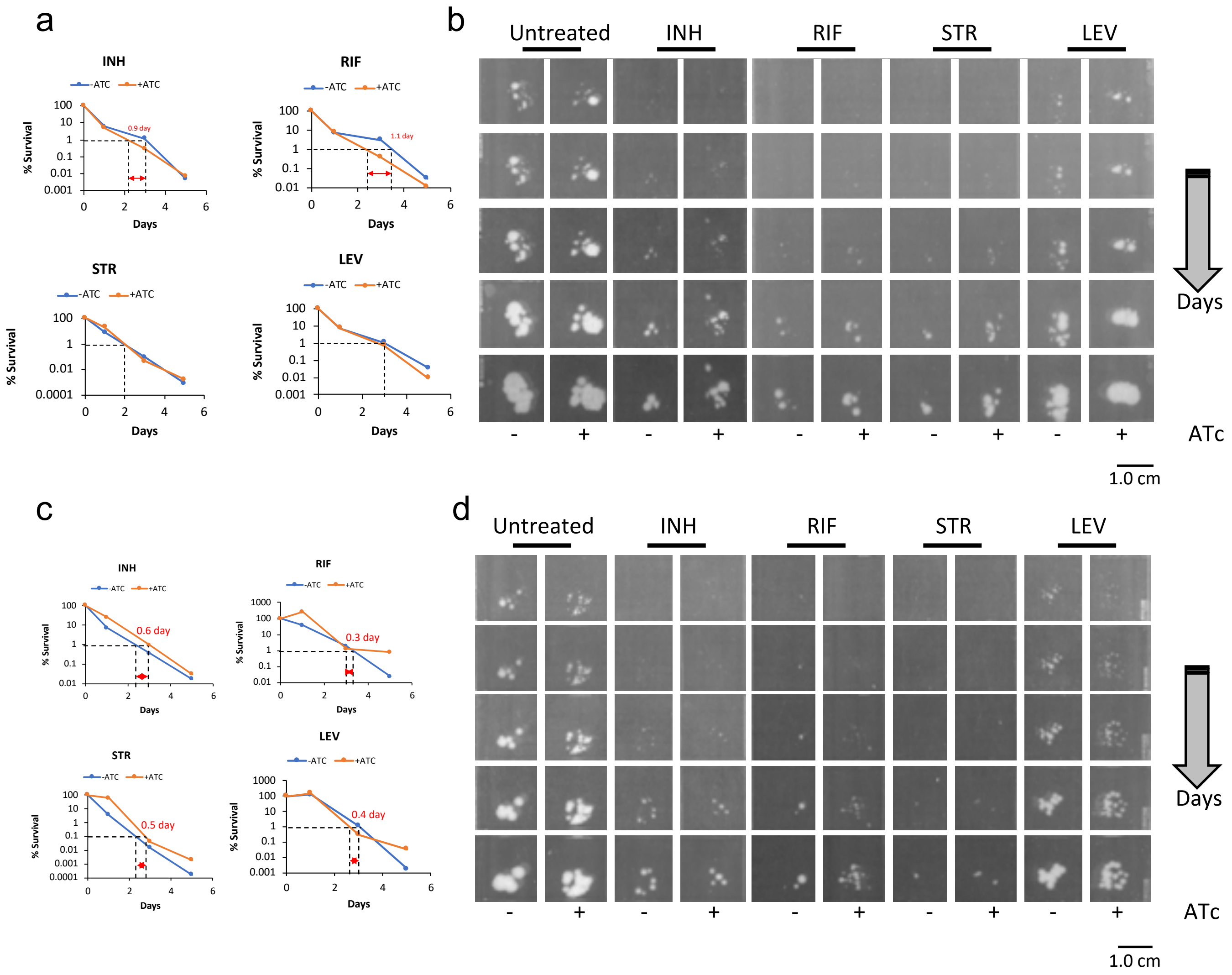

Supplement: Supplementary file 1 — Supplementary Information [file 42003_2023_5059_MOESM1_ESM.pdf]
