# Supplementary material for: ResR/McdR-regulated protein translation machinery contributes to drug resilience in Mycobacterium tuberculosis
Source: Commun Biol. 2023 Jul 11;6:708. doi: 10.1038/s42003-023-05059-8 (PMC10336103; doi:10.1038/s42003-023-05059-8)
Supplement: Supplementary file 2 — Description of Additional Supplementary Data [file 42003_2023_5059_MOESM2_ESM.docx]

**Description of Additional Supplementary Files**

File name: Supplementary Data 1.

Description: Source data for figures.

File name: Supplementary Data 2.

Description: List of differentially regulated genes in resR/mcdR(-).

File name: Supplementary Data 3.

Description: Analysis of ResR/McdRrecognition sequence in the upstream regions of its regulons (AATNACA-N4-TGTNATT with 2 mismatches in either arm)

File name: Supplementary Data 4.

Description: List of oligos used in this study.
