# Supplementary material for: ResR/McdR-regulated protein translation machinery contributes to drug resilience in Mycobacterium tuberculosis
Source: Commun Biol. 2023 Jul 11;6:708. doi: 10.1038/s42003-023-05059-8 (PMC10336103; doi:10.1038/s42003-023-05059-8)
Supplement: Supplementary file 7 — Reporting Summary [file 42003_2023_5059_MOESM7_ESM.pdf]

nature portfolio

Corresponding author(s): Nisheeth Agarwal

Last updated by author(s): 14/06/2023

Reporting Summary

Nature Portfolio wishes to improve the reproducibility of the work that we publish. This form provides structure for consistency and transparency in reporting. For further information on Nature Portfolio policies, see our Editorial Policies and the Editorial Policy Checklist.

Statistics

For all statistical analyses, confirm that the following items are present in the figure legend, table legend, main text, or Methods section.

n/a	Confirmed	
☐	☒	The exact sample size (n) for each experimental group/condition, given as a discrete number and unit of measurement
☐	☒	A statement on whether measurements were taken from distinct samples or whether the same sample was measured repeatedly
☐	☒	The statistical test(s) used AND whether they are one- or two-sided<br>*Only common tests should be described solely by name; describe more complex techniques in the Methods section.*
☒	☐	A description of all covariates tested
☒	☐	A description of any assumptions or corrections, such as tests of normality and adjustment for multiple comparisons
☐	☒	A full description of the statistical parameters including central tendency (e.g. means) or other basic estimates (e.g. regression coefficient) AND variation (e.g. standard deviation) or associated estimates of uncertainty (e.g. confidence intervals)
☐	☒	For null hypothesis testing, the test statistic (e.g. F, t, r) with confidence intervals, effect sizes, degrees of freedom and P value noted<br>*Give P values as exact values whenever suitable.*
☒	☐	For Bayesian analysis, information on the choice of priors and Markov chain Monte Carlo settings
☒	☐	For hierarchical and complex designs, identification of the appropriate level for tests and full reporting of outcomes
☒	☐	Estimates of effect sizes (e.g. Cohen's d, Pearson's r), indicating how they were calculated

Our web collection on statistics for biologists contains articles on many of the points above.

Software and code

Policy information about availability of computer code

Data collection	No software was used for data collection.
Data analysis	Data were analyzed with the help of GraphPad Prism v 7.0e software.

For manuscripts utilizing custom algorithms or software that are central to the research but not yet described in published literature, software must be made available to editors and reviewers. We strongly encourage code deposition in a community repository (e.g. GitHub). See the Nature Portfolio guidelines for submitting code & software for further information.

Data

Policy information about availability of data

All manuscripts must include a data availability statement. This statement should provide the following information, where applicable:

- Accession codes, unique identifiers, or web links for publicly available datasets
- A description of any restrictions on data availability
- For clinical datasets or third party data, please ensure that the statement adheres to our policy

The RNASeq raw data is available from the GEO database under project accession number GSE214729

Human research participants

Policy information about studies involving human research participants and Sex and Gender in Research.

Reporting on sex and gender	Not relevant in our study
Population characteristics	Not relevant in our study
Recruitment	Not relevant in our study
Ethics oversight	Not relevant in our study

Note that full information on the approval of the study protocol must also be provided in the manuscript.

Field-specific reporting

Please select the one below that is the best fit for your research. If you are not sure, read the appropriate sections before making your selection.

☒ Life sciences ☐ Behavioural & social sciences ☐ Ecological, evolutionary & environmental sciences

For a reference copy of the document with all sections, see nature.com/documents/nr-reporting-summary-flat.pdf

Life sciences study design

All studies must disclose on these points even when the disclosure is negative.

Sample size	Four samples/time point/group were used in animal infection experiments. Three samples/strain were used in RNASeq experiment. Six samples/strain were used for analysis of metabolites.
Data exclusions	No data exclusion.
Replication	All data analyses are reproducible.
Randomization	Randomization is not relevant in our study.
Blinding	Blinding is not relevant in our study

Reporting for specific materials, systems and methods

We require information from authors about some types of materials, experimental systems and methods used in many studies. Here, indicate whether each material, system or method listed is relevant to your study. If you are not sure if a list item applies to your research, read the appropriate section before selecting a response.

Materials & experimental systems

n/a	Involved in the study	
☐	☒	Antibodies
☒	☐	Eukaryotic cell lines
☒	☐	Palaeontology and archaeology
☐	☒	Animals and other organisms
☒	☐	Clinical data
☒	☐	Dual use research of concern

Methods

n/a	Involved in the study	
☒	☐	ChIP-seq
☒	☐	Flow cytometry
☒	☐	MRI-based neuroimaging

Antibodies

Antibodies used	Anti-ResR antibodies were commercially raised in rabbit using ResR specific peptide, ELASRRKHRDRKIG by Genscript (www.genscript.com), and anti-puromycin antibodies- Clone 4G11, were obtained from Merck .
Validation	Anti-ResR antibodies were validated by ELISA using specific peptide. Lack of signal in the absence of the peptide indicated its specificity. Anti-puromycin was validated by Western Blotting of HEK293 cell lysates treated with puromycin and cyclohexamide or with Puromycin only. Relatively poor signals with the puromycin and cyclohexamide treated samples indicated its specificity to puromycinylated proteins.

Animals and other research organisms

Policy information about studies involving animals; ARRIVE guidelines recommended for reporting animal research, and Sex and Gender in Research

Laboratory animals	BALB/c mince were used for animal infection experiment.
Wild animals	n/a
Reporting on sex	8-9 weeks old female mice were used for infection, as there are evidences that female mice are more resistant to disease than male counterpart (PMID 24722144)
Field-collected samples	n/a
Ethics oversight	n/a

Note that full information on the approval of the study protocol must also be provided in the manuscript.
